# Continuous flow asymmetric synthesis of chiral active pharmaceutical ingredients and their advanced intermediates†

**DOI:** 10.1039/d1gc01615f

**Published:** 2021-08-03

**Authors:** Sándor B. Ötvös, C. Oliver Kappe

**Affiliations:** Institute of Chemistry, University of Graz, NAWI Graz Heinrichstrasse 28 A-8010 Graz Austria oliver.kappe@uni-graz.at; Center for Continuous Flow Synthesis and Processing (CC FLOW), Research Center Pharmaceutical Engineering GmbH (RCPE) Inffeldgasse 13 A-8010 Graz Austria

## Abstract

Catalytic enantioselective transformations provide well-established and direct access to stereogenic synthons that are broadly distributed among active pharmaceutical ingredients (APIs). These reactions have been demonstrated to benefit considerably from the merits of continuous processing and microreactor technology. Over the past few years, continuous flow enantioselective catalysis has grown into a mature field and has found diverse applications in asymmetric synthesis of pharmaceutically active substances. The present review therefore surveys flow chemistry-based approaches for the synthesis of chiral APIs and their advanced stereogenic intermediates, covering the utilization of biocatalysis, organometallic catalysis and metal-free organocatalysis to introduce asymmetry in continuously operated systems. Single-step processes, interrupted multistep flow syntheses, combined batch/flow processes and uninterrupted one-flow syntheses are discussed herein.

## Abstract 2

This review surveys continuous flow approaches for the synthesis of chiral APIs and their advanced stereogenic intermediates, covering the utilization of biocatalysis, organometallic catalysis and metal-free organocatalysis to introduce asymmetry.

## Graphical abstract

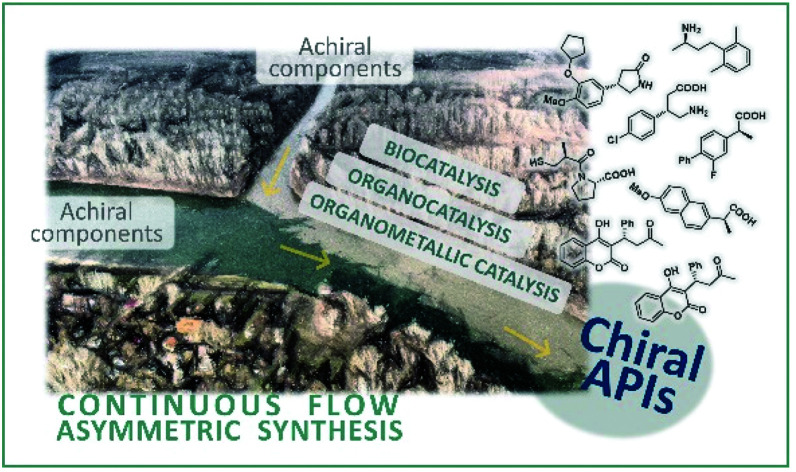

## 1. Introduction

**Fig. 1:**
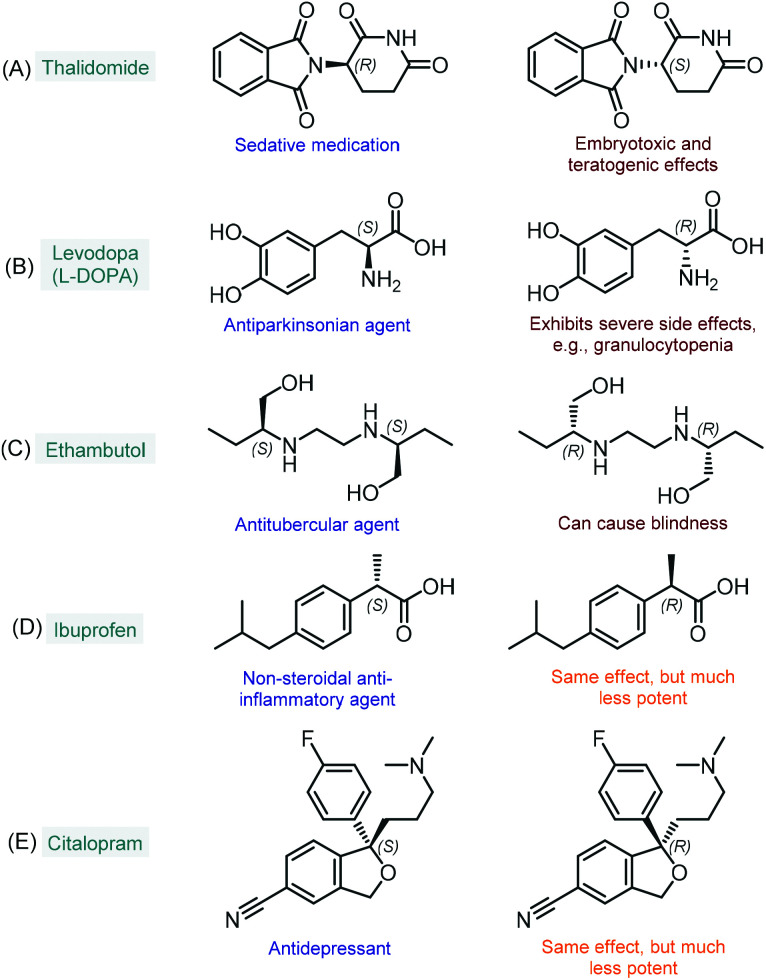
Examples for chiral APIs with significant differences in biological activities of the enantiomers.

The enantioselective synthesis of chiral molecules plays an outstanding role in the pharmaceutical industry.^1^ This can primarily be explained by the fact that the majority of drugs contain chiral molecules as active pharmaceutical ingredients (APIs) and that individual enantiomers of these substances may behave differently under physiological conditions.^2^ As a result of stereospecific interactions with biological systems, distinct enantiomers may demonstrate significant differences in pharmacokinetic, pharmacodynamic and toxicological properties.^2*a*^ In many cases, one enantiomer of a chiral API exerts the desired biological activity, whereas the other one displays qualitatively different biological actions; in extreme cases, it may even be toxic. The most infamous representative of this category is without doubt thalidomide, which was first marketed in the late 1950s as a sedative medication in its racemic form.^3^ It soon became clear, however, that unlike the therapeutic (*R*)-enantiomer, (*S*)-thalidomide exerts embryotoxic and teratogenic effects (Fig. 1A).^4^ Another example is levodopa (or l-DOPA), a well-known antiparkinsonian agent which is marketed in its single l-enantiomer form (*S* absolute configuration) due to severe side effects of the d-isomer (Fig. 1B).^5^ Similarly, the antitubercular ethambutol is a single-enantiomer medication with the (*S*,*S*)-isomer being responsible for the desired therapeutic activity, while the (*R*,*R*)-enantiomer can cause blindness due to optical neuritis (Fig. 1C).^6^ In numerous cases, only one enantiomer of a chiral API is biologically active, while the other isomer are either inactive or bear the same activity, but to a comparatively small extent. In these instances, although the ineffective isomers do not necessarily involve harmful side effects, their presence is unproductive and these substances may therefore be regarded as impurities.^7^ For example, the non-steroidal anti-inflammatory agent, ibuprofen is typically formulated as a racemic mixture; however, its (*S*)-enantiomer is over 100-fold more potent as an inhibitor of cyclooxygenase 1 enzyme than the (*R*)-isomer (Fig. 1D).^8^ Similarly, the (*S*)-enantiomer of the antidepressant citalopram (escitalopram) is much more potent than the (*R*)-form; however, the racemate is also available as a commercial medication (Fig. 1E).^9^ As compared to the previous cases, it is relatively scarce that stereoisomers of chiral APIs are equipotent. For instance, certain antimalarials (such as, halofantrine and mefloquine) as well as antiarrhythmic agents (such as, mexiletine, propafenone and flecainide) exhibit small or no differences in the potency of their enantiomers.^10^

The application of enantiomerically pure drugs implies obvious benefits as concerns therapeutic, toxic and pharmacokinetic effects, while in many cases it also enables the administration of lower dosages as compared with the corresponding racemic substances.^11^ Additionally, when therapeutic activity is associated with only one enantiomer, the gratuitous existence of the ineffective isomers is a direct source for various environmental burdens; for example, concerning the environmental fate and ecotoxicity of the substances involved.^12^ In fact, the most recent guidelines of the U.S. Food and Drug Administration (FDA) and the European Medicines Agency (EMA) on the development of chiral drugs strongly support single-enantiomers over racemic compounds.^13^ Consequently, most new chiral drugs are single enantiomers,^14^ and numerous chiral drugs that had hit the market earlier as racemates were recently switched to a more potent single-enantiomer version.^15^

One of the pivotal challenges of drug manufacturing is derived from the inherent complexity of APIs. Typical manufacturing schemes require multistep synthetic protocols of numerous and diverse chemical transformations, which demand individual reaction control, optimization, work-up, purification and analytical techniques for each segment included.^1^ In case of manufacturing of enantiomerically pure APIs, the challenges are amplified further with the introduction of the appropriate chiral information.^16^ Classical approaches typically utilize chiral auxiliaries or naturally occurring homochiral building blocks from the chiral pool to introduce asymmetry,^16*b*,17^ or widely employ various resolution techniques to separate racemates.^16*a*,18^ These approaches generally ensure excellent enantiomeric purities; however, chiral pool strategies are strongly limited by the molecular diversity of the parent compounds, whereas separation of enantiomers *via* resolution requires special instrumentation (*e.g.*, chiral chromatography) and/or additional chemical and physical manipulations, such as diastereoisomeric salt formation and crystallization. In contrast, enantioselective synthesis takes advantage of a well-defined chiral environment in the form of a chiral catalyst or a ligand, and provide a more direct and atom economic access to certain *enantio*-enriched products using readily available achiral components.^19^

Pharmaceutical manufacturing is currently among the most polluting chemical fields.^20^ Complex synthetic procedures of APIs typically involve *E*-factors of >100,^21^ which translates to the generation of at least 100 kg of chemical waste during the production of each kilogram of a pharmaceutical substance. It is important to recognize that chemical processes can not only be advanced by new and improved chemical transformations, but also *via* strategic utilization of enabling technologies.^22^ I this manner, continuous flow chemistry has provoked an enormous attention during the last two decades.^23^ This processing strategy offers exceptional opportunities to accelerate, integrate, simplify, scale-up and automatize chemical reactions and has acquired a great importance in the context of green and sustainable syntheses.^24^ As amply demonstrated, typical continuous flow setups furnish novel process windows and increased parameter spaces for process intensification,^25^ and allow chemical transformations to be performed with extraordinarily control over the most important reaction conditions due to the greatly enhanced heat and mass transfer and improved mixing properties.^23^ The inherent technical benefits of continuous flow equipment facilitate on-demand generation and simultaneous consumption of toxic or highly reactive reagents, which would otherwise be impossible under conventional batch conditions.^26^ These features open up novel reaction pathways within traditionally forbidden chemical spaces,^27^ and imply not only higher reaction rates and improved selectivity,^28^ but also safer and greener chemistries.^29^ Importantly, continuous flow reaction technologies ensure inherent scalability without re-optimization of critical reaction parameters,^30^ and allow multistep reactions to be combined into telescoped one-flow sequences without isolation of any intermediates.^31^ This permits a simplified and scalable access to complex target substances and involves significantly less purification and work-up issues and thus the generation of less waste.^32^ In addition, by exploiting in-line process analytical technologies (PATs) and real-time analytical feedback-driven reaction control, uninterrupted multistep synthesis lines can be operated as integrated systems while maintaining optimum conditions.^33^ With expanding economical and societal demands on sustainability, quality and costs, pharmaceutical industry is gradually realizing the concept of continuous flow manufacturing. At the same time, the FDA and the EMA are intensively exploring the potentials of this technology,^34^ and most major pharma-companies are testing or already implementing flow chemistry-based technologies either for process development or for manufacturing purposes.^35^

In recent years, continuous flow processes involving enantioselective catalysis have attracted considerable attention due to their significant advantages over the corresponding batch reactions, such as lower catalyst loading, higher selectivities, shorter reaction times as well as higher productivity and facile scalability. Both homogeneous as well as immobilized chiral catalysts have been found useful in continuous flow systems.^36^ As concerns practical and environmental aspects, the application of supported catalysts in fixed-bed flow systems are especially appealing.^36*g*,*k*,*l*^ However, in some cases leaching of catalytic species may limit direct scalability and result the contamination of the chiral product. In contrast, homogeneous catalytic systems offer very simple and reliable operations, but these typically cannot be recycled and require waste-consuming purification steps to remove.

During the past few years, continuous flow asymmetric catalytic strategies have reached maturity and started to extend from development of new synthetic concepts to target-oriented applications. As such, continuous flow enantioselective synthesis of chiral APIs is a rapidly emerging field which attracts an upsurge of continuous interest in academia and in industry. To the best of our knowledge, this field has not yet been surveyed in a specialized review before. Therefore, in the present study we are aiming to give a detailed picture on continuous flow processes in which chiral APIs and their advanced intermediates are accessed *via* asymmetric catalytic transformations as key steps. The scope of this review comprises active ingredients responsible for the direct medical effect of actual marketed drugs or pharmacologically active substances which were investigated in at least in stage I clinical trials. Reactions involving biocatalysis, organometallic catalysis as well as metal-free organocatalysis will be considered utilizing either homogeneous or heterogeneous catalytic sources. Special emphasis will be placed onto aspects related to green chemistry and process sustainability. Contributions in which chiral auxiliaries are employed to introduce asymmetry or in which the racemic form of a chiral medication is prepared are not discussed in detail herein.^37^ Also, asymmetric hydrogenations are excluded from the scope of the present study due to the availability of specialized recent reviews on that topic.^38^ In the following three sections, contributions on continuous flow asymmetric synthesis of chiral APIs and their advanced intermediates are categorized on the basis of the nature of chiral catalysis employed in the key synthetic steps to introduce asymmetry.

## 2. Organometallic asymmetric catalysis

Enantioselective catalysis enabled by chiral coordination complexes is one of the foremost strategies to access enantioenriched products.^39^ As demonstrated by the pioneering works of Sharpless, Knowles and Noyori on enantioselective transition metal-catalyzed oxidations and hydrogenations, and their Nobel Prize in Chemistry in 2001, truly outstanding achievements have been attained in this field.^40^ The toolbox of metal-mediated asymmetric transformations have considerably been expanded in the past decades, and has become a well-established strategy for the manufacture of enantioenriched drugs and pharmaceutical products.^41^

**Scheme 1:**
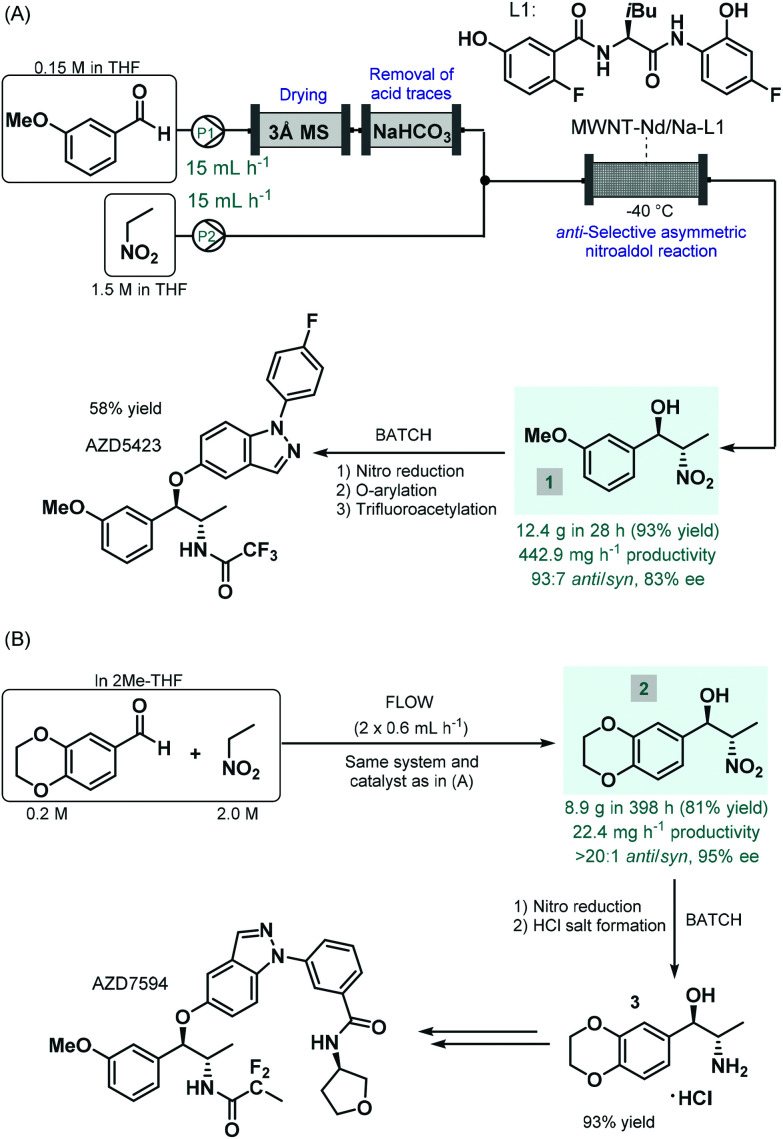
Synthesis of chiral key intermediates of phase II experimental drugs under combined flow and batch reaction conditions.^42,47^

The first report on a chiral coordination complex-catalyzed asymmetric C–C bond forming reaction employed for the synthesis of an advanced API intermediate under flow conditions was published by Hashimoto, Kumagai and Shibasaki in 2014.^42^ The *anti*-selective asymmetric nitroaldol reaction between *m*-methoxybenzaldehyde and nitroethane was investigated in order to yield a chiral precursor of AZD5423 (Scheme 1A), a phase II experimental drug developed for the treatment of chronic obstructive pulmonary disease (COPD).^43^ Notably, the achievement of *anti*-diastereoselectivity was proven as a significant synthetic challenge in nitroaldol reactions due to the *syn*-preferred chelation models of most earlier catalytic systems.^44^ The authors therefore developed a novel Nd/Na heterobimetallic complex comprising a chiral amide-based ligand with appropriate spatial arrangement ensuring high *anti*-diastereoselectivity.^45^ In order to achieve a robust heterogeneous material applicable under continuous flow conditions, the catalyst was supported within a multiwalled carbon nanotube (MWNT) matrix *via* self-assembly of the chiral ligand and the corresponding metal salts.^46^ A simple packed-bed flow setup was assembled consisting of separate reactant streams. In order to remove any moisture as well as acidic impurities which could reduce catalyst lifetime, the aldehyde feed was passed through sequential scavenger columns containing molecular sieves and solid NaHCO_3_. After initial small-scale runs, the flow synthesis was scaled-up using a larger catalyst column. Within a processing time of 28 h, 12.4 g (93% yield) of the corresponding chiral β-nitro alcohol (**1**) was achieved with an *anti*/*syn* ratio of 93 : 7 and with an ee of 88%. Compound **1** was then transformed into AZD5423 in batch *via* nitro reduction, copper-mediated *O*-arylation and trifluoroacetylation with 58% yield. Notably, by using the heterogenous catalytic flow process, the work-up and isolation of the chiral key intermediate could largely be simplified compared to the corresponding batch reaction. However, due to the lack of strong covalent immobilization forces, some leaching of catalytic species was observed. The Shibasaki group later expanded the utility of the MWNT-immobilized Nd/Na heterobimetallic complex-catalyzed *anti*-selective asymmetric nitroaldol reaction for the flow synthesis of a chiral intermediate of AZD7594 (Scheme 1B),^47^ another potent drug candidate (currently in phase II clinical development) for the treatment of asthma and COPD.^48^ The reaction of 1,4-benzodioxane-6-carbaldehyde and nitroethane was investigated in a similar flow setup demonstrated in the earlier study. In this case, the synthesis proved less productive than in the previous example; it afforded 8.9 g (81% yield) of chiral adduct **2** (*anti*/*syn* ratio 20 : 1, ee 95%) in *ca.* 400 h, which corresponded to a productivity of 22.4 mg h^−1^. The nitro group in compound **2** was subsequently reduced under conventional batch conditions and treated with a mixture of HCl/MeOH to yield the HCl salt form of the corresponding amino alcohol (**3**), as chiral key intermediate of AZD7594.

**Scheme 2:**
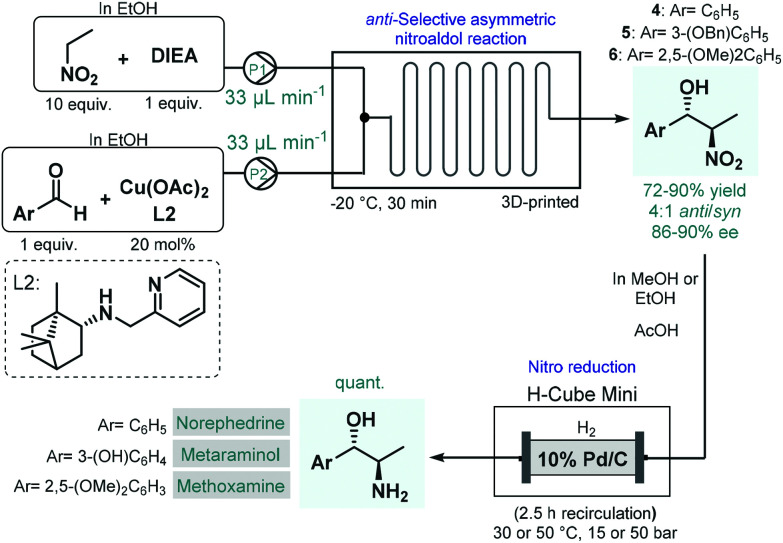
Asymmetric flow synthesis of sympathomimetic amines, norephedrine, metaraminol and methoxamine.^49^

In 2017, Benaglia and co-workers exploited 3D-printed custom-made flow reactors to perform *anti*-selective asymmetric nitroaldol reactions in the presence of a chiral Cu(ii) complex, and utilized the method for the continuous flow synthesis of sympathomimetic amines, norephedrine, metaraminol and methoxamine (Scheme 2).^49^ Various stereoisomers of these substances exhibit important pharmacological activities and bear diverse medical uses; for example, as decongestant or appetite suppressant, or for the treatment of hypotension, or against low blood pressure. For the nitroaldol reactions, the chiral complex (in 20 mol% with respect to Cu) was generated *in situ* from Cu(OAc)_2_ and a camphor-derived chiral ligand. The best reaction conditions were identified as −20 °C and 30 min residence time, in EtOH as an environmentally-benign solvent. Under these conditions, the corresponding chiral β-nitro alcohols (**4–6**) were obtained in high yields (72–90%) and with good ee's (86–90%); however, diastereoselectivities were only at around 4 : 1 for the *anti*-isomer. In order to obtain the targeted chiral amino alcohols, nitro reductions (in case of metaraminol, also *O*-debenzylation) were next performed under flow conditions using an H-Cube Mini hydrogenation reactor equipped with a cartridge containing 10 wt% Pd/C as catalyst. Although the asymmetric nitroaldol reaction and the subsequent reduction(s) were not telescoped, amino alcohol products were isolated without any intermediate purifications or solvent switching thereby resulting a sustainable methodology. Importantly, the possibility of continuous in-line removal and recycling of the homogeneous chiral catalyst were demonstrated by simple filtration through a short column of silica.

**Scheme 3:**
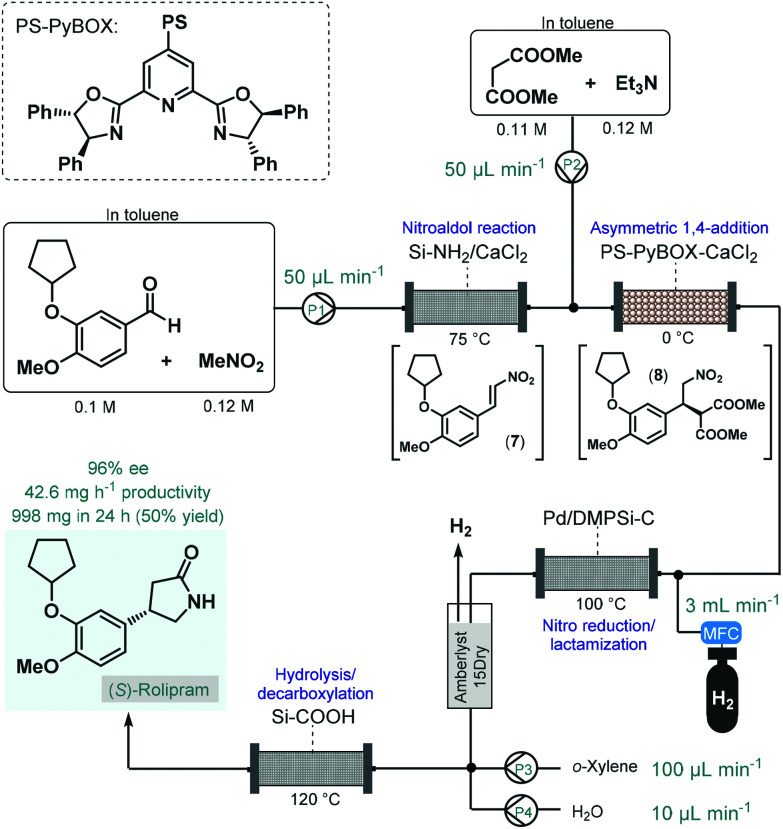
Multistep telescoped continuous flow asymmetric synthesis of (*S*)-rolipram.^50^

In 2015, Kobayashi and co-workers reported a telescoped procedure for the multistep continuous flow enantioselective synthesis of (*R*)- and (*S*)-rolipram using a series of different heterogeneous catalysts in packed-bed columns (Scheme 3).^50^ This pioneering process was the first successful example for multistep one-flow chiral API synthesis using asymmetric catalysis. Rolipram is a derivative of γ-aminobutyric acid (GABA); it is a selective phosphodiesterase-4 inhibitor, which proved useful as anti-inflammatory as well as antidepressant agent in clinical trials.^51^ Despite it has not yet been marketed as a drug, rolipram has numerous activities which attract continuing focus of research. For example, it has been proposed as a treatment for multiple sclerosis, and has been suggested to bear antipsychotic, immunosuppressive and antitumor effects.^52^ The multistep synthetic strategy to access rolipram from readily available achiral components comprised four consecutive catalytic transformations: (1) a nitroaldol reaction with concomitant water elimination, (2) an asymmetric conjugate addition, (3) nitro reduction/lactamization and (4) ester hydrolysis/decarboxylation. In the first step, nitrostyrene **7** was obtained from the corresponding aldehyde and nitromethane while being passed through a column loaded with a mixture of anhydrous CaCl_2_ and a silica-supported amine as base catalyst. In the next step, asymmetry was introduced *via* enantioselective conjugate addition between nitrostyrene **7** and dimethyl malonate at 0 °C in the presence of triethylamine as a base and a polymer-supported chiral calcium catalyst. The catalyst, developed earlier by the same authors,^53^ comprised CaCl_2_ and a polystyrene (PS)-immobilized chiral pyridinebisoxazoline (PyBOX) ligand. It facilitated access to γ-nitro ester **8** with an excellent ee of 94%, which was next subjected to nitro reduction/lactamization *via* hydrogenation in the presence of dimethylpolysilane-modified Pd/C (Pd/DMPSi-C) as catalyst at 100 °C. After in-line purification using a cartridge loaded with Amberlyst 15Dry and H_2_-degassing, the stream containing the chiral lactam was passed through a column containing a silica-supported carboxylic acid to obtain (*S*)-rolipram *via* a hydrolysis/decarboxylation sequence at 120 °C. The telescoped flow system was operated continuously for 24 h and provided 998 mg (50% yield) of (*S*)-rolipram with 96% ee. By simply exchanging the polymer-supported chiral calcium catalyst to its opposing enantiomer, (*R*)-rolipram could readily be synthesized under otherwise identical reaction conditions. Importantly, the system proved stable for extended periods (at least for one week) with no leaching of the metal catalysts. As a result of elimination of intermediate purifications as well as the use of robust and stable heterogeneous catalysts in all synthetic steps, the reported procedure is highly beneficial from environmental aspects. Although, this example points out that the one-flow combination of atom economic synthetic steps enable sustainable production at the lab scale, the direct scalability of the process may be limited due to the high dilutions applied.

**Scheme 4:**
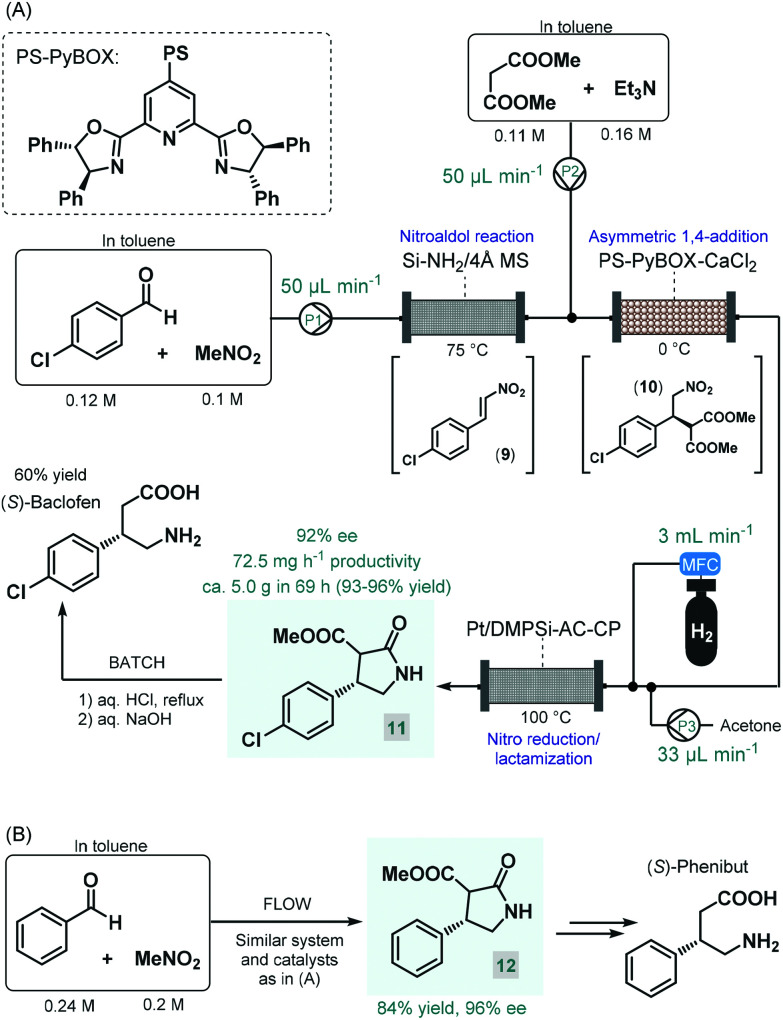
Multistep continuous flow asymmetric synthesis of chiral baclofen and phenibut precursors.^55,57^

Baclofen, a 3-substituted GABA derivative, is an antispastic drug that is widely employed as a muscle relaxant in the case of certain types of spasticity.^54^ A three-step approach, similar to their earlier enantioselective rolipram synthesis, was utilized by the Kobayashi group for the telescoped flow synthesis of a chiral baclofen precursor (Scheme 4A).^55^ In the first step, a mixture of *p*-chlorobenzaldehyde and nitromethane in toluene was passed through a cartridge containing a silica-supported amine and 4 Å MS to obtain nitrostyrene **9***via* nitroaldol condensation. The resulting stream was combined with a toluene solution of dimethyl malonate together with Et_3_N and was then directed through a column charged with PS-supported PyBOX-CaCl_2_ as chiral catalyst to furnish γ-nitro ester **10***via* enantioselective conjugate addition (92% ee). For the subsequent catalytic hydrogenation/nitro reduction, various palladium catalysts afforded poor selectivity, and resulted in dechlorination of the aromatic ring. The authors therefore developed a DMPSi-modified platinum catalyst supported on activated carbon (AC) and calcium phosphate (CP; Pt/DMPSi-AC-CP), which enabled selective nitro reduction without dehalogenation and afforded chiral lactam **11** in high yields. The telescoped flow system was operated continuously for 69 h resulting in approx. 5.0 g of **11** (93–96% yield) with an ee of 92%. The chiral intermediate was converted to (*S*)-baclofen in 60% yield by treatment with aq. HCl and NaOH solutions under batch conditions. The closely related medication, phenibut is used to treat anxiety and insomnia.^56^ Kobayashi and co-workers employed the same strategy for the synthesis of its advanced chiral intermediate (**12**) starting from benzaldehyde and nitromethane *via* similar reaction steps as detailed above (Scheme 4B).^57^

**Scheme 5:**
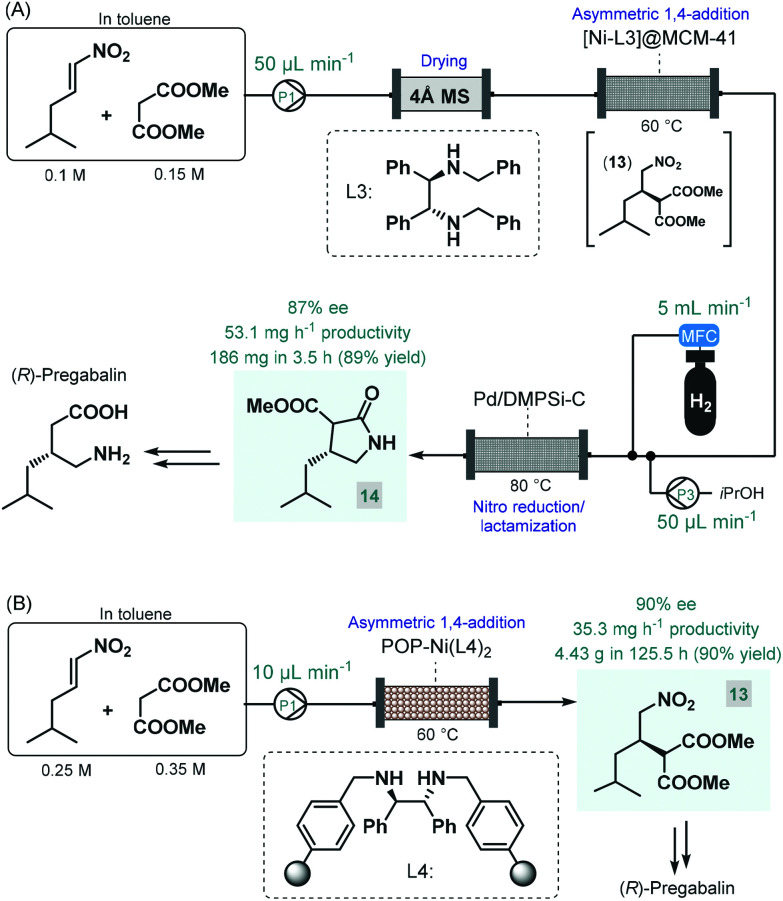
Continuous flow asymmetric synthesis of chiral pregabalin precursors.^59,60^

Pregabalin is another important representative of the family of GABA-derivatives. It is an anxiolytic and anticonvulsant drug employed for treatment of epilepsy, neuropathic pain, fibromyalgia, and generalized anxiety disorder.^58^ Considering the obvious structural similarities with rolipram and baclofen, its asymmetric synthesis relying on an enantioselective dimethyl malonate–nitroalkene conjugate addition seemed plausible following the same synthetic strategy reported for other GABA-derivatives. However, the PS-supported PyBOX-CaCl_2_, used earlier as chiral catalyst in such transformations, demonstrated poor enantioselectivities in reactions of nitroolefins bearing primary aliphatic substituents. Therefore, Kobayashi and co-workers developed a novel composite by deposition of a chiral nickel–diamine complex *via* wet impregnation to a mesoporous aluminosilicate material, MCM-41.^59^ The catalyst thus prepared furnished excellent enantioselectivity in the conjugate addition between dimethyl malonate and the appropriate aliphatic nitroalkene in a continuous flow packed bed system. The resulting chiral adduct (**13**) was transformed further into γ-lactam **14**, a chiral precursor of (*R*)-pregabalin, by means of nitro reduction/lactamization in the presence of H_2_ gas and Pd/DMPSi-C as heterogeneous catalyst. During a 3.5 h run, the two-step telescoped flow process afforded 186 mg (89% yield) of **14** with an ee of 87% (Scheme 5A). The heterogeneous catalyst used in this study relied on non-covalent forces for immobilization of the chiral ligand thereby resulting in leaching and limited recyclability. Therefore, Kramer and co-workers later took a different approach and covalently immobilized a similar nickel–bisdiamine complex on a polystyrene porous organic polymer (POP), where due to the strong covalent interactions, almost no leaching of the chiral complex occurred.^60^ The authors successfully utilized the heterogenous chiral complex as catalyst for asymmetric conjugate addition of malonates to various aliphatic nitroalkenes under batch conditions. Importantly, a packed-bed setup was assembled and the synthesis of chiral pregabalin intermediate **13** was investigated under flow conditions by performing the appropriate conjugate addition (Scheme 5B). The flow system was operated continuously for more than five days producing 4.43 g (90% yield) of **13** in 90% ee. Notably, the Kobayashi research group also prepared the racemic version of pregabalin precursor **14***via* a three-step telescoped continuous flow Knoevenagel condensation-1,4-addition-nitro reduction/lactamization sequence.^37*f*^ In addition, Seeberger and co-workers reported a multistep flow assembly system with interchangeable reaction modules for the synthesis of racemic pregabalin, phenibut, baclofen and rolipram.^37*g*^

**Scheme 6:**
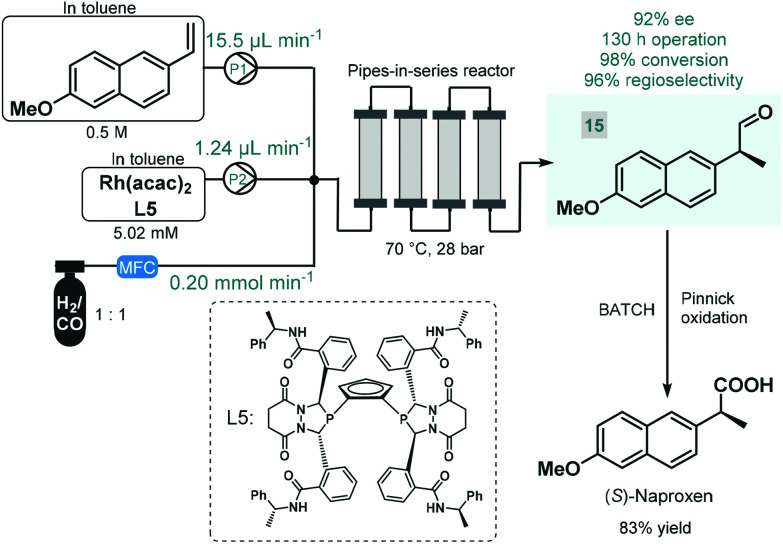
Continuous flow enantioselective synthesis of a chiral naproxen precursor.^62^

Continuous flow reactors have emerged as a useful platform for the efficient, safe and scalable utilization of gases in chemical synthesis.^61^ Although asymmetric hydrogenations have successfully been applied for the synthesis of chiral pharmaceuticals under continuous flow conditions,^38^ the application of further gas–liquid transformations for asymmetric flow API synthesis is scarcely exemplified. In fact, we found only one contribution. In 2016, Landis and co-workers reported the enantioselective hydroformylation of 2-vinyl-6-methoxynaphthalene catalyzed by a chiral Rh-bisdiazaphospholane complex in a tubular flow reactor leading to a key chiral intermediate (**15**) of the nonsteroidal anti-inflammatory drug (*S*)-naproxene (Scheme 6).^62^ A pipes-in-series-type reactor consisting of a small diameter segmented flow regime as well as numerous vertical columns were used for the study, which was designed to ensure residence times in the region of 0.5–12 h.^63^ Toluene solutions of the chiral catalyst and the substrate along with syngas (CO/H_2_ 1 : 1) were fed separately into the reactor, and the effects of the most important reaction conditions were carefully investigated to achieve high conversion and high ee while maintaining good regioselectivity. The reactor was operated for a total of 130 h, and the chiral aldehyde (**15**) was obtained in multigram quantities and with 92% ee. Subsequently, (*S*)-naproxene was obtained by Pinnick oxidation of intermediate **15** under conventional batch conditions. As concerns safety and environmental points, although a non-recyclable homogeneous catalyst was employed in this example, its loading was kept relatively low; whilst the possibility to safely handle syngas proved as a further benefit compared with the corresponding batch reaction.

## 3. Chiral organocatalysis

From practical and environmental aspects, asymmetric transformations using metal-free organocatalysts are exceptionally attractive and this approach has opened up new possibilities for stereoselective transformations.^64^ The first important milestone in asymmetric organocatalysis was the l-proline-catalyzed enantioselective intramolecular aldol reaction of cyclic triketones (Hajos–Parrish–Eder–Sauer–Wiechert reaction) in the 1970s;^65^ however, the potential of this finding remained unexplored for almost three decades. The revival of this chemistry is closely related with the discovery of the proline-catalyzed direct asymmetric intermolecular aldol and Mannich reactions in the early 2000s.^66^ Since then, various amino acids, phosphoric acids, alkaloids, and their well-tailored derivatives have become useful as organocatalysts for numerous asymmetric carbon–carbon and carbon–heteroatom bond forming reactions.^67^ Due to their beneficial features, such as molecular diversity, broad scope, non-toxicity and stability under various reaction conditions, chiral organocatalysts have proven significant utility for the synthesis of chiral drugs and other enantioenriched bioactive products.^68^

**Scheme 7:**
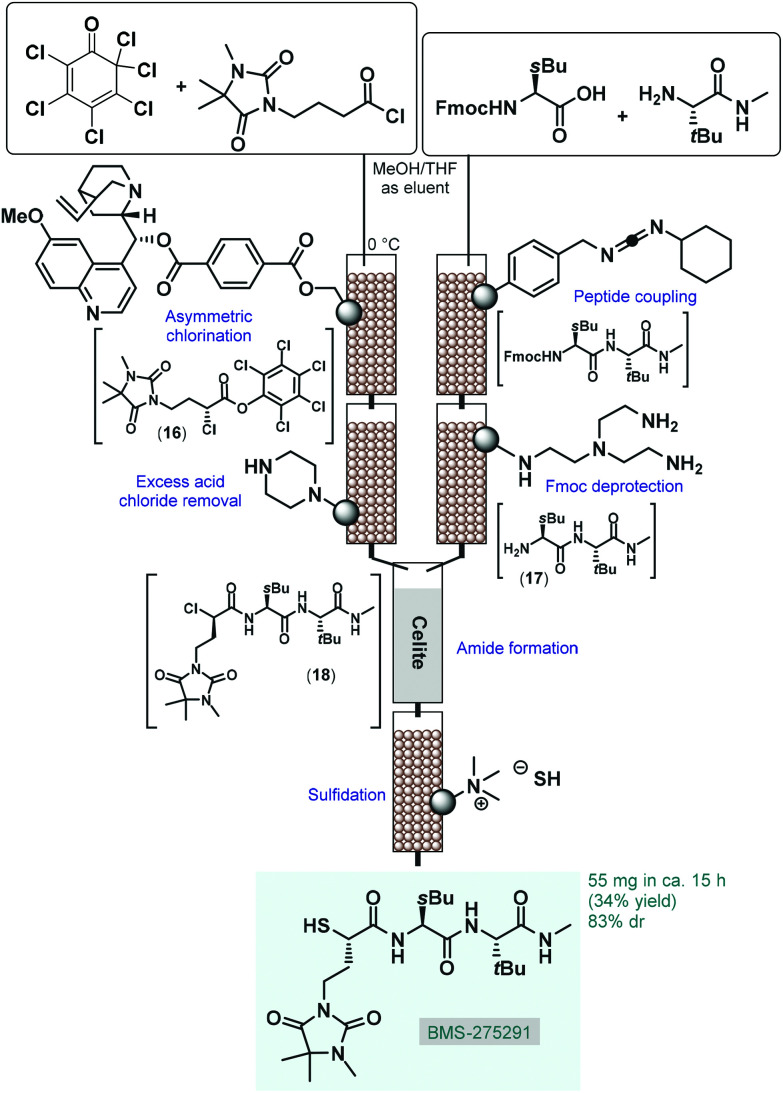
Diastereoselective synthesis of an earlier drug candidate, BMS-275291 on sequentially linked catalyst and reagent columns.^69^

Most processes for continuous flow asymmetric catalytic synthesis of chiral APIs and their advanced intermediates have been published during the last five years. However, in 2005 Lectka and co-workers demonstrated an early example employing a gravity-driven flow through approach on sequentially linked catalyst and reagent columns for the diastereoselective synthesis of a metalloproteinase inhibitor, BMS-275291 (Scheme 7).^69^ This compound was actively investigated in stage III clinical trials for the treatment of non-small cell lung cancer, however studies were later discontinued.^70^ The key step of the multistep synthesis was an asymmetric halogenation reaction catalyzed by quinine-functionalized Wang resin beads.^71^ The cinchona alkaloid served as a stoichiometric base for dehydrohalogenation as well as a chiral catalyst for asymmetric chlorination. The resulting mixture containing α-chloroester **16** was passed through a scavenger column containing a piperazino-functionalized resin to remove excess acid chloride. In a parallel stream, the appropriate peptide fragment (**17**) of the target substance was prepared by successive peptide coupling and Fmoc deprotection on sequential carbodiimide- and tris-(2-aminoethyl)amine-functionalized columns. The streams containing dipeptide **17** and α-chloroester **16** were next combined on a column loaded with Celite to give α-chloroamide **18**, which was finally passed through another column loaded with a solid SH^−^ source (sulfide-exchanged Amberlite-400 resin) to yield BMS-275291 *via* sulfidation. Similarly to other related studies of the authors,^72^ catalysts and reagents were simply placed in a series of jacketed addition funnels as fixed-bed columns, and substrates were continuously percolating through by means of gravity in the presence of an appropriate carrier solvent. The whole procedure took around 15 h and resulted 55 mg (34% yield) of the desired product with 83% diastereomeric excess (de). Although the processes exhibited important advantages over the corresponding batch synthesis (*e.g.*, shorter reaction time, simpler product isolation and facile catalyst recovery), the most obvious limitation of such semi-continuous gravity-driven flow through approaches is that without precisely set fluid flow rates and residence times, the reproducibility of the experiments is comparatively low. Moreover, solid supported reagents enabled simple purification with less amount of waste generated; however, these solid substances required frequent regeneration or replacement preventing truly scalable and environmentally reliable continuous operations. In spite of these shortcomings, these pioneering findings shed light onto the great potentials of solid supported organocatalysts in the continuous flow synthesis of complex chiral substances.

**Scheme 8:**
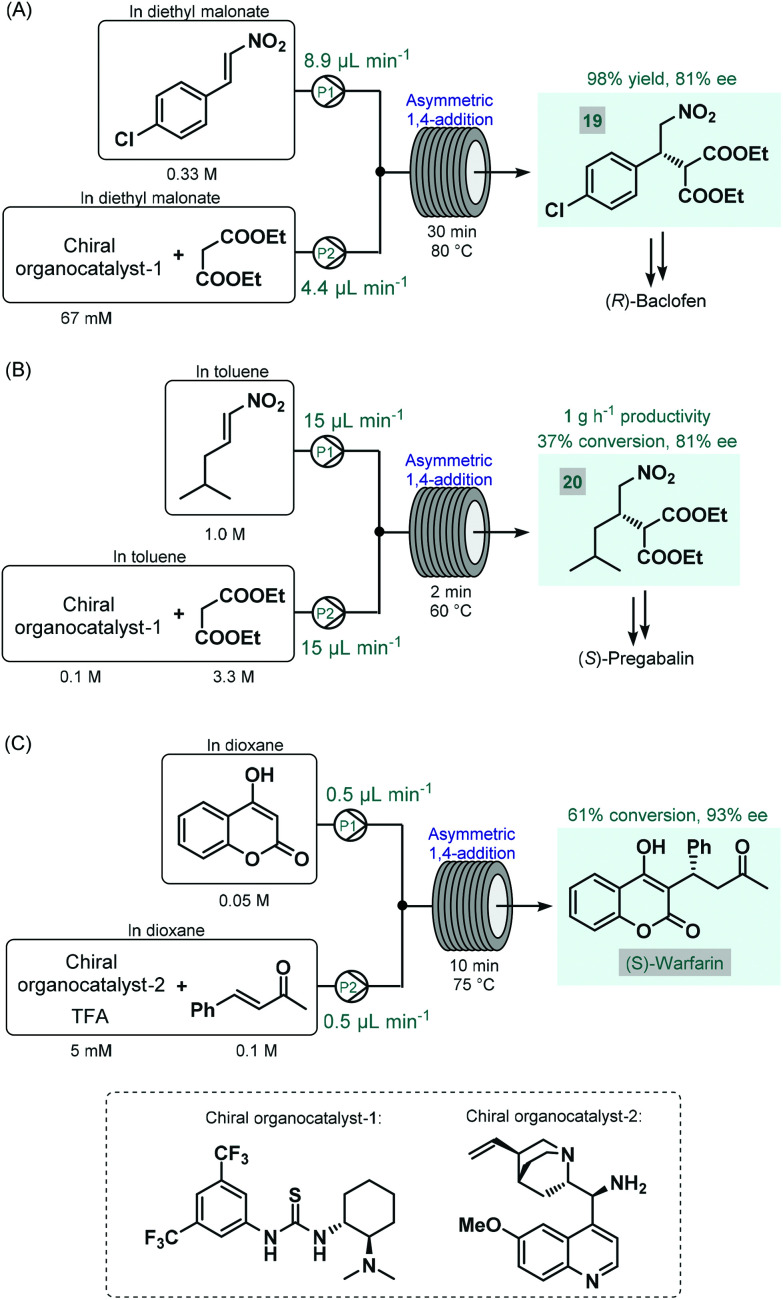
Enantioselective synthesis of GABA-derived API precursors as well as (*S*)-warfarin in homogenous organocatalytic flow processes.^73^

The first reports on enantioselective organocatalytic syntheses of chiral APIs or their advanced intermediates under continuous flow conditions were reported by Benaglia and co-workers in 2015.^73^ In these studies, the authors employed soluble organocatalysts in glass microreactors or in heated reaction coils. Initially, the synthesis of an advanced baclofen precursor was investigated by performing an enantioselective conjugate addition between *p*-chloro-β-nitrostyrene and diethyl malonate in the presence of a bifunctional thiourea-organocatalyst (Scheme 8A).^73*a*^ An excess of diethyl malonate served as a reaction medium without the need for an additional solvent. The effects of the most important reaction conditions were rapidly explored using a 10 μL microreactor. Under optimum conditions (30 min residence time and 80 °C), scale-up was performed in a larger reaction coil, and the targeted baclofen precursor (**19**) was achieved in 98% yield and with 81% ee. The authors later applied a similar flow approach for the enantioselective synthesis of an (*S*)-pregabalin precursor (**20**) and also of the anticoagulant, (*S*)-warfarin.^73*b*^ The enantioselective synthesis of pregabalin precursor **20** was accomplished by conjugate addition of diethylmalonate to the corresponding aliphatic nitroalkene in the presence of the same chiral thiourea-catalyst used in their earlier study (Scheme 8B). Although under the best reaction conditions (2 min residence time and 60 °C) the reaction furnished only 37% conversion, the chiral precursor was obtained in good productivity of 1 g h^−1^ and with 81% ee. For the asymmetric synthesis of warfarin, the enantioselective Michael addition of 4-hydroxycoumarin to benzylideneacetone was investigated in the presence of a cinchona alkaloid-derived chiral primary amin catalyst and trifluoroacetic acid (TFA) as a co-catalyst (Scheme 8C). Good results (61% conversion, 93% ee) were achieved within 10 min residence time using a 10 μL microreactor at 75 °C; however, during attempts to scale-up in a larger coil, a significant drop in conversion occurred, possibly because of mixing issues. The same Michael addition was later studied by Belder co-workers in a microreactor combined with an integrated 2D-HPLC-MS chip to facilitate the online monitoring of the ee.^74^ In each experiment, a small portion of the reaction mixture exiting the microreactor was transferred to the analytical chip by an injection valve. In this manner, the on-chip device enabled rapid optimization of warfarin synthesis with minimal amounts of material being consumed and minimal amounts of waste being produced.

**Scheme 9:**
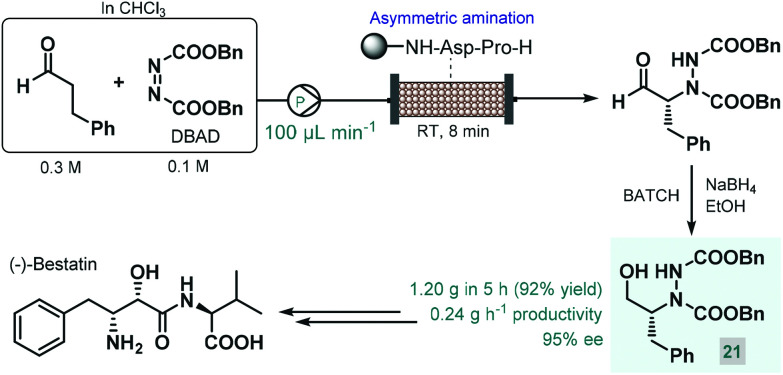
Continuous flow enantioselective synthesis of a key (−)-bestatin intermediate using a solid supported peptide as chiral organocatalyst.^75^

**Scheme 10:**
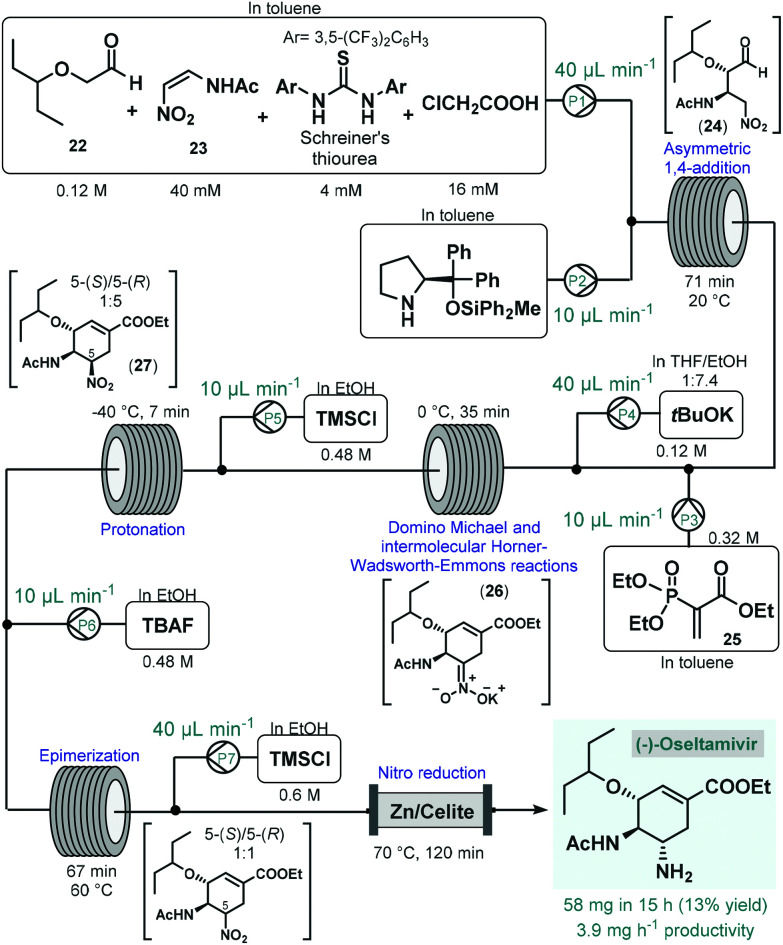
Five-step telescoped continuous flow asymmetric organocatalytic synthesis of (−)-oseltamivir.^82^

**Scheme 11:**
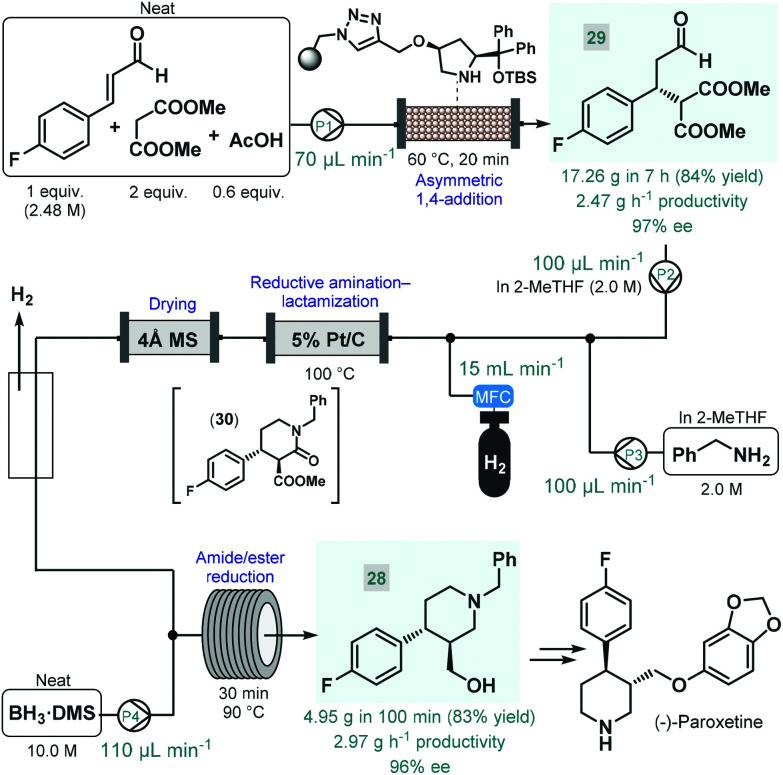
Continuous flow enantioselective synthesis of the key phenylpiperidine intermediate of (−)-paroxetine.^86^

In 2015, Fülöp and co-workers developed a continuous flow process for the enantioselective α-amination of aldehydes with dibenzyl azodicarboxylate (DBAD) as electrophilic nitrogen source using a solid-supported prolyl-peptide as chiral organocatalyst.^75^ Earlier, the same reaction was explored by the Pericàs group using a resin-supported diphenylprolinol silyl ether as catalyst under batch and flow conditions; however, pre-treatment of the catalyst was necessary with a large excess of the aldehyde to prevent its deactivation by the azodicarboxylate component.^76^ The α-hydrazino aldehydes obtained as product in these reactions have proven useful as chiral precursors in the synthesis of various biologically active substances, including natural products and APIs.^68*a*,*b*,*d*^ In the study by Fülöp and co-workers,^75^ the asymmetric reaction of 3-phenylpropanal with DBAD followed by NaBH_4_ reduction directly resulted a chiral key intermediate (**21**) of (−)-bestatin (Scheme 9), a potent aminopeptidase and enkephalinase inhibitor that has been studied in clinical trials for the treatment of various forms of cancer.^77^ After 5 h of continuous operation, the organocatalytic reaction furnished 1.20 g (92% yield) of the corresponding α-hydrazino alcohol (**21**) with an excellent ee of 95%. The configurationally labile stereocenter of the α-hydrazino aldehyde product may be enolized by the secondary amine moiety of the catalyst. The authors therefore pointed out the importance of precise residence time control in achieving high ee and high conversion at the same time.

(−)-Oseltamivir is a neuraminidase inhibitor which is broadly employed for the treatment of influenza.^78^ Importantly, due to its broad-spectrum antiviral activity, it was clinically investigated against SARS-CoV-2 ^79^ in various in clinical trials.^80^ Although, there are numerous synthetic routes developed,^78^ the asymmetric synthesis of (−)-oseltamivir is challenging due the presence of three contiguous stereocenters within the cyclohexene ring. Continuing their long-standing interest in the synthesis of this medication,^81^ the Hayashi group reported the continuous flow telescoped synthesis of (−)-oseltamavir in 2017 (Scheme 10),^82^ which was in fact the first multistep one-flow process involving enantioselective organocatalysis as key step to introduce asymmetry. The five-step process was based on their earlier achievements on the one-pot batch synthesis of (−)-oseltamivir with further optimizations (*e.g.*, shortening of reaction times, finding alternative solvents to eliminate clogging) required to implement the synthesis under flow conditions.^81*a*,*b*^ In the first flow unit, asymmetric conjugate addition of aldehyde **22** to (*Z*)-*N*-2-nitroethenylacetamide (**23**) resulted chiral key intermediate **24** in the presence of a diphenylprolinol silyl ether-type homogeneous organocatalyst combined with Schreiner's thiourea and chloroacetic acid as additives. This step was achieved within 71 min residence time in toluene under dilute conditions, as polar solvents resulted low yields due to quick epimerization to the undesired *anti*-adduct. In the next coil, domino Michael reaction and intermolecular Horner–Wadsworth–Emmons reactions took place. For this, phosphoryl acrylate **25** and a solution of *t*BuOK in THF/EtOH were fed *via* two consecutive streams furnishing cyclic intermediate **26** at 0 °C after 35 min residence time. In the following step, protonation of the nitronate anion with HCl, formed *in situ* from trimethylsilyl chloride (TMSCl), resulted the corresponding nitro compound (**27**) within 7 min residence time at −40 °C. As in the protonation step the undesired 5-(*R*)-isomer was formed predominantly, in the next unit epimerization was performed within 67 min residence time at 60 °C using tetrabutylammonium fluoride (TBAF) in EtOH. Finally, reduction of the nitro group was achieved in the presence of TMSCl while being passed through a heated column filled with a mixture of zinc powder and Celite within 2 h residence time. The system was operated continuously for 15 h to furnish 58 mg of pure (−)-oseltamivir after work-up and purification, which corresponded to 13% yield with a total residence time of 310 min. In this example, a complicated five-step synthesis was accomplished in a telescoped manner thereby eliminating intermediate purifications and reducing waste formation and process time considerably. On the downside however, the productivity of the process was low which limits potential larger-scale applications. Notably, Watts and Sagindra recently reported a ten-step semi-telescoped flow process for the synthesis of (−)-oseltamivir phosphate starting from shikimic acid as a readily available chiral building block.^83^

(−)-Paroxetine is a selective serotonin reuptake inhibitor used for the treatment of depression, anxiety and panic disorder.^84^ In most of the reported synthesis routes, phenylpiperidine **28** serves as chiral key intermediate and gives the final API *via* simple etherification and N-deprotection.^85^ Our research group reported a highly productive continuous flow approach for the catalytic enantioselective synthesis of **28** (Scheme 11).^86^ The key step to introduce asymmetry was an asymmetric conjugate addition between 4-fluorocinnamaldehyde and dimethyl malonate in the presence of a polystyrene-supported *cis*-4-hydroxydiphenylprolinol *tert*-butyldimethylsilyl (TBS) ether as chiral organocatalyst, developed earlier by Pericàs and co-workers as a modified version of classical *trans* analogues.^87^ Importantly, the conjugate addition was achieved under solvent-free conditions by pumping a neat mixture of the aldehyde, 2 equiv. of the malonate and some acetic acid as additive through a heated catalyst column within 20 min residence time. The flow system was operated continuously for 7 h to obtain 17.26 g (84% yield) of chiral aldehyde **29** (97% ee) after removing unreacted components by evaporation, thus furnishing an outstanding productivity of 2.47 g h^−1^ of pure product. Despite the solvent-free conditions applied, the supported organocatalyst proved highly robust and ensured constant selectivity and conversions in the range of 85–93% during the long-run experiment. Importantly, the flow process generated minimal amounts of waste as indicated by an *E*-factor of only 0.7 and resulted in a significant reduction of the effective catalyst loading compared with the corresponding batch reaction. The chiral adduct was processed further *via* a telescoped reductive amination–lactamization–amide/ester reduction sequence utilizing heterogeneous catalytic hydrogenation followed by neat BH_3_·dimethylsulfide (DMS) complex-mediated reductions. Both steps were designed to avoid formation of large amounts of metallic waste and to enable compatibility with larger scale flow operations. Solutions (2.0 M each) of chiral aldehyde **29** and benzylamine in 2-MeTHF were fed separately, and after being combined with H_2_ gas, the mixture was passed through a heated column containing 5% Pt/C as hydrogenation catalyst. The resulting stream containing the desired *trans* lactam (**30**) as major product (*trans*/*cis* 93 : 7) was directed through a 4 Å MS column to remove water traces released during reductive amination and to prevent decomposition of BH_3_·DMS downstream. After removing excess H_2_ through a buffer flask, the dried and degassed stream was combined with neat BH_3_·DMS, and amide/ester reduction took place during passage through a heated coil. Notably, neat BH_3_·DMS is prone to intensive thermal decomposition, and may evolve pyrophoric B_2_H_6_ and H_2_ gases upon contact with moisture; however, it could safely be handled under controlled flow conditions.^88^ After a 100 min long-run, the telescoped process furnished 4.95 g (83% yield) of enantiomerically enriched phenylpiperidine **28** (96% ee), which corresponded to a productivity of 2.97 g h^−1^. The whole process, starting from 4-fluorocinnamaldehyde and dimethyl malonate, involved a cumulative *E*-factor of 6.22 with the bio-derived 2-MeTHF as the only solvent applied making this approach attractive from environmental aspects.

**Scheme 12:**
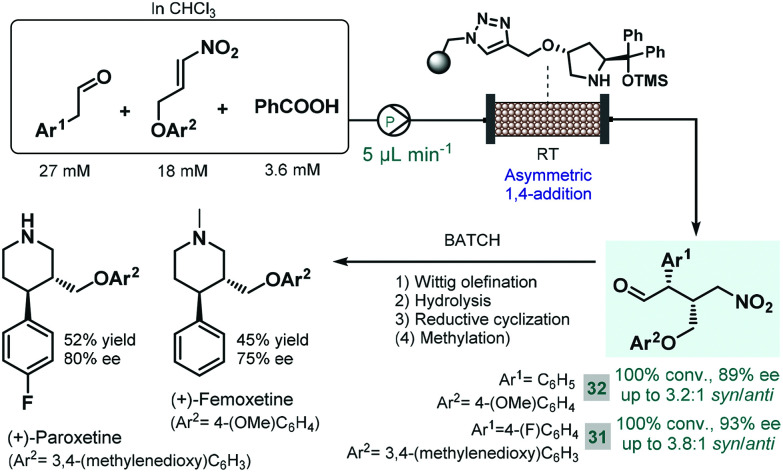
Combined batch and flow synthesis of (+)-paroxetine and (+)-femoxetine.^89^

Later Szcześniak and co-workers investigated asymmetric Michael additions of phenylacetaldehydes to nitroalkanes to achieve chiral intermediates for the synthesis of (+)-paroxetine and the closely related (+)-femoxetine.^89^ Initially, the reactions were performed under batch conditions by using a commercially available diphenylprolinol trimethylsilyl (TMS) ether as soluble organocatalyst. Despite achieving good conversions and high ee's, diastereoselectivities were low (*syn*/*anti* 1.3 : 1 or 1.2 : 1) due to epimerization of the configurationally labile α-substituted γ-nitroaldehyde products. Considering that epimerization was caused by the organocatalyst itself, the authors next employed continuous flow conditions using the immobilized version of the catalyst with the aim to fine-tune the contact time necessary for the conjugate addition and to minimize epimerization simultaneously (Scheme 12). By pumping a dilute reaction mixture containing the appropriate aldehyde and nitroalkane together with benzoic acid as an additive at 5 μL min^−1^ flow rate, diastereoselectivity was improved to around 3.0 : 1 (*syn*/*anti*), while obtaining high conversions. The chiral adducts (**31** and **32**) achieved in the flow process were transformed into (+)-paroxetine and (+)-femoxetine *via* consecutive Wittig olefination, hydrolysis and reductive cyclization (as well as methylation for femoxetine) under batch conditions.

**Scheme 13:**
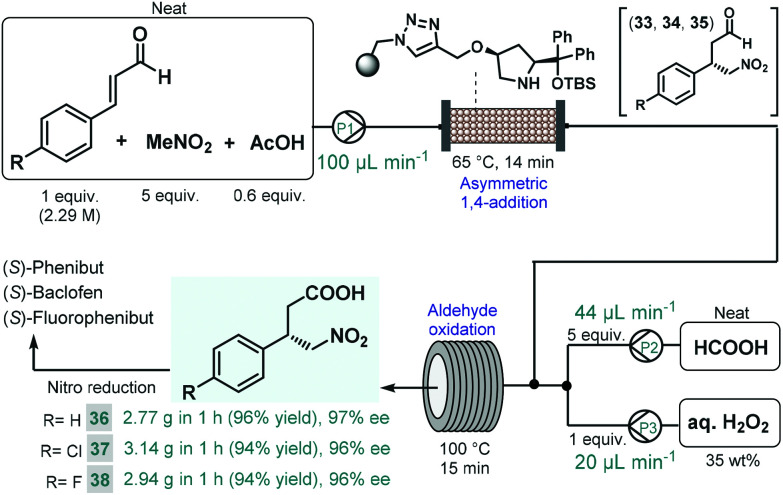
Continuous flow synthesis of chiral γ-nitrobutyric acids as key intermediates of baclofen, phenibut, and fluorophenibut.^90^

We recently reported a telescoped continuous flow process for the synthesis of optically active γ-nitrobutyric acids as key intermediates of the GABA analogues baclofen, phenibut, and fluorophenibut (Scheme 13).^90^ The first step of the synthesis comprised the enantioselective Michael-type addition of nitromethane to the appropriate cinnamaldehyde derivatives in the presence of the same solid-supported *cis*-4-hydroxydiphenylprolinol-type organocatalyst that was used earlier in our multistep paroxetine-precursor synthesis.^86^ The neat reaction mixture containing 1 equiv. of the corresponding cinnamaldehyde, 5 equiv. of nitromethane and 0.6 equiv. of acetic acid as additive was pumped continuously through a heated column (65 °C, 14 min residence time) packed with the heterogeneous organocatalyst to furnish chiral γ-nitroaldehydes **33**, **34** and **35**. The subsequent aldehyde oxidation was accomplished by taking advantage of an *in situ* performic acid generator utilizing formic acid as a benign precursor of the potentially explosive oxidant.^91^ Neat formic acid and an aqueous H_2_O_2_ solution were thus pumped at flow rates that corresponded to 1 equiv. of H_2_O_2_ and 5 equiv. of formic acid with respect to the aldehyde stream exiting the organocatalyst column. After combining all three streams, the resulting mixture was passed through a reaction coil where simultaneous performic acid generation and aldehyde oxidation took place within 15 min residence time at 100 °C. In this manner, the potentially dangerous oxidizing agent was safely formed and consumed *in situ* within a closed reactor environment. The telescoped system was operated continuously for 1 h in all three cases, and furnished γ-nitrobutyric acids **36**, **37** and **38** in high yields (up to 96%) and with ee's up to 97%. The two-step process enabled productivities up to 3.14 g h^−1^ of pure products after simple isolation by evaporation. Importantly, the methodology exploited a highly reusable heterogenous catalyst and relied on an environmentally benign and inexpensive oxidant under neat conditions, thus generating small amount of waste as demonstrated by cumulative *E*-factors of around 2.

**Scheme 14:**
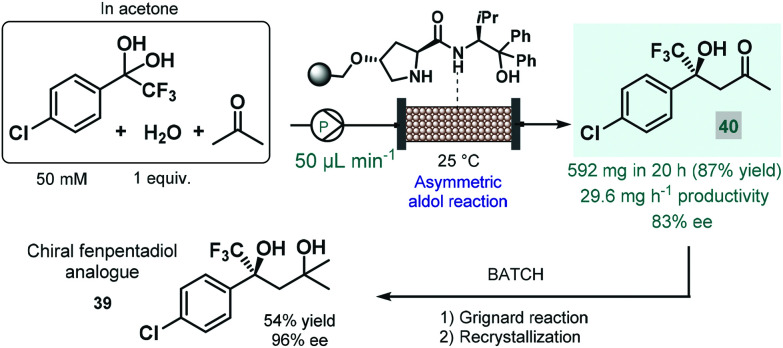
Continuous flow enantioselective synthesis of a chiral fenpentadiol analogue.^92^

In 2021 Kobayashi, Yamashita and Yue reported an efficient approach for enantioselective aldol reactions of trifluoroacetophenones with ketones under flow conditions using a polystyrene-supported prolinamide as chiral organocatalyst.^92^ The reaction was initially investigated under batch conditions where the inherent reversibility of the aldol reaction lead to racemization and thus insufficient enantioselectivities over a prolonged reaction time. In contrast, under flow conditions the continuous substrate stream was contacting with a high local catalyst excess but for a comparably shorter residence time, thereby minimizing the racemization phenomenon and thus warranting high ee's. It was also observed that the presence of water as an additive was necessary to promote hydrolysis of the catalytic iminium intermediate and thus to achieve reasonably long catalyst lifetimes (>195 h). The process was successfully applied for the synthesis of a chiral fenpentadiol analogue (**39**; Scheme 14), exhibiting potential antidepressant activity. For this, trifluoromethylated chiral ketone **40** was obtained *via* enantioselective aldol reaction under optimized flow conditions utilizing acetone as an environmentally sound solvent, and was next converted to the corresponding tertiary alcohol in Grignard reaction with methyl magnesium bromide under batch conditions.

**Scheme 15:**
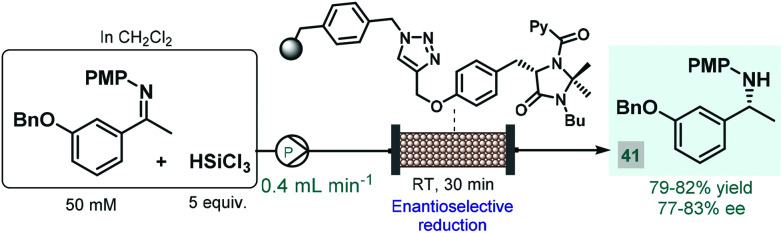
Synthesis of a chiral rivastigmine precursor *via* continuous flow enantioselective reduction.^93^ (PMP = *p*-methoxyphenyl).

In 2017, the Benaglia group reported a solid supported chiral *N*-picolylimidazolidinone as an efficient heterogeneous organocatalyst for the enantioselective, metal-free reduction of imines with trichlorosilane.^93^ Under batch conditions, the immobilized catalyst exhibited facile reusability and showed activity and selectivity comparable with its homogeneous counterpart reported earlier by the same group.^94^ Encouraged by these results, the authors employed the heterogeneous *N*-picolylimidazolidinone catalyst in a packed-bed flow system in order to synthesize 1-(*m*-benzyloxyphenyl)-ethylamine, a valuable chiral precursor of various APIs, such as rivastigmine, a cholinesterase inhibitor used for the treatment of Alzheimer's disease (Scheme 15).^95^ During a 6 h run followed by treatment with aq. NaOH solution and extractive work-up, chiral amine **41** was obtained in 79–82% yield with 77–83% ee. The same authors employed a related trichlorosilane-mediated reduction strategy utilizing various homogeneous chiral picolinamide catalysts for batch and flow synthesis of advanced intermediates of the antiparkinsonian agent rasagiline as well as tamsulosin (used for the treatment of prostatic hyperplasia).^96^ In these cases, modest enantioselectivities were achieved with the chiral organocatalysts, therefore easily removable chiral auxiliaries were employed as elements of stereocontrol to obtain the desired enantiomerically pure amino compounds.^97^

## 4. Enantioselective biocatalysis

Biocatalytic transformations have also emerged as powerful means to access to enantiomerically pure substances from prochiral precursors.^98^ The most important benefit of biocatalysis is that natural enzymes are capable of promoting highly chemo-, regio- and stereoselective transformations under reasonably mild conditions. The enhanced selectivity enables more straightforward and more sustainable syntheses and generates less waste. However, in most cases, the generality and flexibility of biocatalytic reactions are limited compared to chemocatalysts due to highly specific substrate–enzyme interactions.^99^ Continuous flow biocatalytic approaches have received an upsurge of interest as highlighted by numerous comprehensive reviews.^100^ This is due to the fact that flow chemistry has been proven as an invaluable tool in overcoming the most important limitations of conventional enzymatic catalysis, such as long reaction times, enzyme instability and incompatibility, tedious work-up and purification procedures as well as difficulties in scale-up and process intensification. These benefits rendered continuous flow biocatalysts particularly appealing for the synthesis of various APIs and important chiral intermediates.

**Scheme 16:**
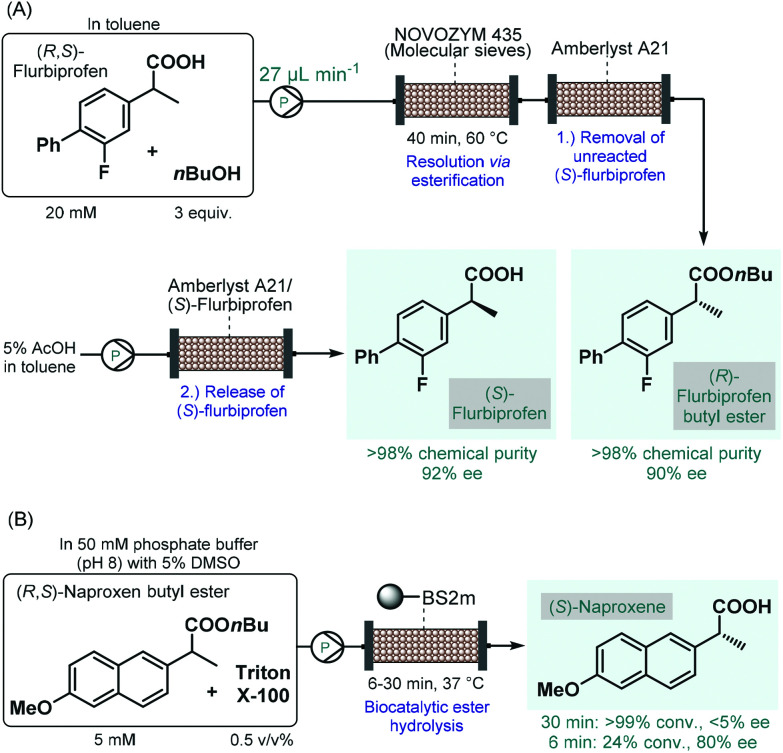
Continuous flow biocatalytic synthesis of chiral propionic acid-derived APIs.^101,108^ (BS2M = esterase from *Bacillus subtilis*).

The earliest examples on continuous flow biocatalysis for the synthesis of chiral APIs reported enzymatic resolutions of racemic active ingredients by using immobilized enzymes in packed-bed flow reactors. For example, in 2012, Conti and co-workers published an efficient protocol for the lipase-catalyzed kinetic resolution of (*R*,*S*)-flurbiprofen,^101^ an important propionic acid derivative, the (*R*)-enantiomer of which is a nonsteroidal anti-inflammatory agent,^102^ while the (*S*)-compound was shown to exhibit anticancer activity.^103^ Immobilised lipase B from *Candida antarctica* (Novozym 435) was reported to bear good enantiopreference toward (*R*)-flurbiprofen in resolution of the racemic compound *via* esterification.^104^ A mixture of (*R*,*S*)-flurbiprofen and *n*-butanol in toluene as solvent was therefore pumped through a column charged with Novozym 435 together with molecular sieves (Scheme 16A). In order to trap unreacted (*S*)-flurbiprofen and to reduce waste formation, a second cartridge loaded with a supported base (Amberlyst A21) was installed downstream. In this manner, the solution exiting the reactor contained (*R*)-flurbiprofen butyl ester with high chemical purity (>98%) and with an ee of 90%. By washing with acetic acid solution, unreacted (*S*)-flurbiprofen could simply be released from the scavenger column and was obtained in high purity (>98%) and with an ee of 92% without further work-up and purification steps needed. Importantly, the flow process required a residence time of 40 min, which is a significant improvement as compared with the batch reaction time of 6 h required for the same reaction. Considering that enzyme purification is known as a time-consuming and expensive procedure, the application of whole cells or lyophilized cells instead of purified enzymes is an appealing approach.^105^ The same group therefore reported a modified procedure for the kinetic resolution of (*R*,*S*)-flurbiprofen *via* enantioselective esterification catalyzed by immobilized-dry mycelia of *Aspergillus oryzae* as a whole cell microbial system.^106^

In the above instances, conversion rates were negatively affected by water traces necessitating in-line water removal by using molecular sieves. In contrast, the enantioselective hydrolysis of ester derivatives of such propionic acids can be achieved in aqueous solutions,^107^ resulting directly the pharmaceutically active free carboxylic acids in a technically simpler and more scalable approach. Accordingly, Paradisi and co-workers studied the enantioselective hydrolysis of racemic flurbiprofen, naproxen and ibuprofen esters in the presence of various esterases in aqueous solutions.^108^ In preliminary batch investigations, the best results in terms of conversion and enantioselectivity were achieved in the resolution of naproxen butyl ester using an engineered esterase from *Bacillus subtilis* covalently immobilized on an agarose support. To overcome solubility issues under flow conditions, a non-ionic surfactant, Triton X-100 was employed in the substrate solution. In this manner, complete hydrolysis of naproxen butyl ester could be achieved at 5 mM concentration within 30 min residence time; however, enantioselectivity was below 5% (Scheme 16B). Importantly, although the conversion decreased with the residence time, the ee could significantly be improved at lower residence times (*e.g.*, 80% ee at 6 min). In these examples, despite the use of the heterogeneous enzymes significantly improves process sustainability, the high dilutions applied may imply high solvent consumption and limit direct scalability.

**Scheme 17:**
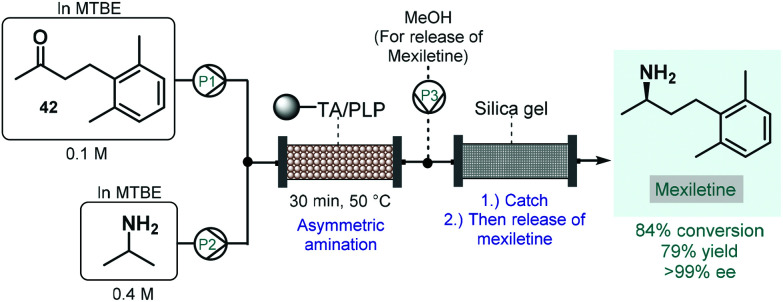
Synthesis of mexiletine *via* continuous flow biocatalytic asymmetric amination.^109^ (TA = ω-transaminase from *Arthrobacter*).

Numerous natural products and APIs contain chiral amine moieties. Jamison and co-workers therefore developed an efficient continuous flow process for the synthesis of chiral amines *via* asymmetric amination of ketones catalyzed by an immobilized transaminase.^109^ Whole cells of *Escherichia coli* expressing the (*R*)-selective ω-transaminase from *Arthrobacter* were immobilized onto a polymeric resin. Unlike, for example, lipases, ω-transaminases are cofactor-dependent enzymes. This means that for the desired biocatalytic activity, the presence of pyridoxal 5′-phosphate (PLP) is necessary as a cofactor enabling the transfer of amino groups from amine donors to ketone groups. Therefore, together with the transaminase, PLP was also immobilized on the carrier, and the resulting complex material was loaded into a reactor column. To suppress leaching of the transaminase or PLP from the catalyst bed, methyl *tert*-butyl ether (MTBE) was chosen as solvent, which is known not to dissolve either of the components of the catalytic material. The flow asymmetric amination was directly utilized for the enantioselective synthesis of mexiletine, a chiral drug used as antiarrhythmic, antimyotonic, and analgesic agent (Scheme 17).^110^ For this, an MTBE solution of ketone **42** and isopropylamine as amino donor was fed separately to the packed-bed reactor heated at 50 °C, where the transamination yielded 84% of the enantiopure mexiletine (ee > 99%) within 30 min residence time. Under these conditions, the system was operated continuously for 5 days with the same enantioselectivity and with only a small loss of enzyme activity (<10%). In order to facilitate product isolation and to minimize waste formation, a scavenger cartridge loaded with silica gel was installed downstream, catching the chiral amine continuously and then releasing it offline by washing with methanol.

**Scheme 18:**
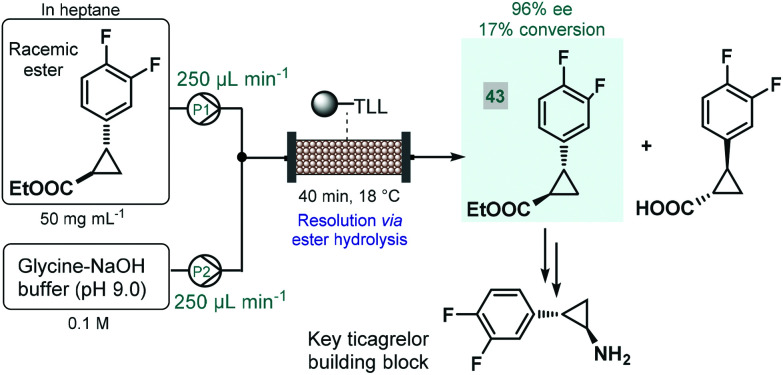
Continuous flow synthesis chiral ester **43***via* biocatalytic resolution.^111^ (TLL = lipase from *Thermomyces lanuginosus*).

**Scheme 19:**
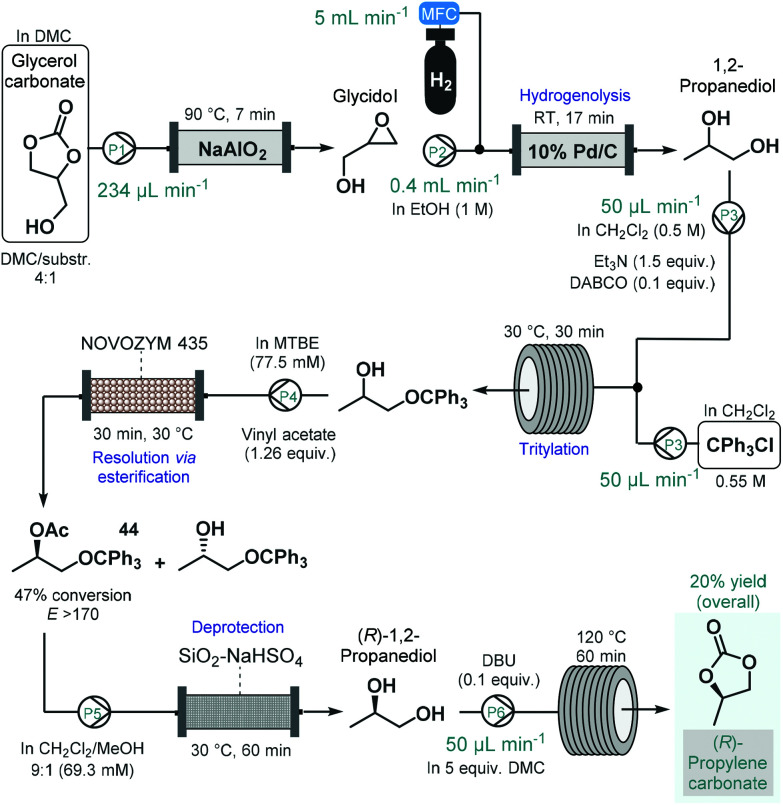
Step-wise continuous flow synthesis of (*R*)-propylene carbonate using a chemoenzymatic resolution as key step to introduce asymmetry.^114^ (DABCO = 1,4-diazabicyclo[2.2.2]octane, DBU = 1,8-diazabicyclo[5.4.0]undec-7-ene).

Biocatalytic approaches have proven useful for the asymmetric synthesis of chiral building blocks of various APIs. For instance, Turner and co-workers reported the lipase-catalyzed kinetic resolution of a cyclopropanecarboxylate ester,^111^ which is a precursor of a chiral key building block of ticagrelor, a chiral drug approved for the treatment of acute coronary symptoms (Scheme 18).^112^ Lipase from *Thermomyces lanuginosus* was immobilized covalently on a polymeric resin and was charged into a stainless steel column as catalyst bed. The separate feds containing the racemic ester and an aqueous buffer solution was combined and was then pumped through the catalyst bed within 40 min of total residence time (recirculation through multiple runs). The targeted (1*R*,2*R*)-ester (**43**) was obtained with an ee of 96% and with an increased productivity compared with the corresponding batch experiment.

(*R*)-Propylene carbonate is a valuable chiral intermediate in the synthesis of various tenofovir pro-drugs which are employed widely for the treatment of HIV and hepatitis B.^113^ Recently, de Souza and co-workers reported a continuous flow process for the synthesis of (*R*)-propylene carbonate with a lipase-mediated kinetic resolution as a key step to introduce asymmetry (Scheme 19).^114^ The synthesis employed glycerol carbonate as starting material which was readily achieved earlier from glycerol, an inexpensive and renewable material.^115^ Glycerol carbonate was transformed into glycidol in the presence of NaAlO_2_ as catalyst in a heated reactor column, which was subsequently converted into 1,2-propanediol by means Pd/C-catalyzed hydrogenolysis. To improve chiral recognition and selection in the subsequent biocatalytic resolution, 1,2-propanediol was tritylated on the primary hydroxyl group. The protected 1,2-propanediol-derivatives were then subjected to kinetic resolution in the presence of Novozym 435 using MTBE as solvent and vinyl acetate as acyl donor to yield the desired enantiomerically enriched protected secondary alcohol (**44**) with high conversion (47%) and with an excellent enantiomeric ratio (*E* > 170).^116^ Importantly, the biocatalytic flow system was operated continuously for 8 h without any change in conversion or selectivity. The removal of acetate and trityl groups were subsequently achieved on a column loaded with silica-supported NaHSO_4_,^117^ and finally (*R*)-1,2-propanediol was converted into (*R*)-propylene carbonate in the presence of an organic base and dimethyl carbonate (DMC) as solvent while being passed through a heated reaction coil. The process afforded (*R*)-propylene carbonate in an overall yield of 20%. Although the steps were not telescoped, an inexpensive and renewable starting material was used together with a combination of reusable heterogeneous catalyst which warranted sustainable operation.

**Scheme 20:**
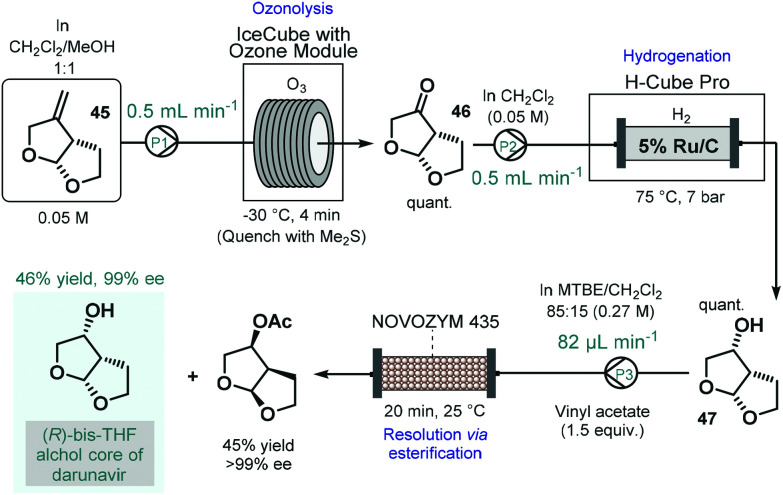
Continuous flow asymmetric synthesis of the bicyclic sidechain of darunavir.^119^

Darunavir is an important antiretroviral agent which is used to treat and prevent HIV.^118^ A three-step continuous flow process was developed by Miranda and co-workers for the asymmetric synthesis of its bicyclic sidechain (Scheme 20).^119^ Alkene **45** was quantitatively converted into the corresponding ketone (**46**) by means of ozonolysis performed in a flow reactor equipped with an ozone generator. The ketone was next submitted to continuous flow reduction in an H-Cube Pro hydrogenation reactor using 5 wt% Ru/C as catalyst to obtain alcohol **47** in a quantitative yield. Finally, the kinetic resolution of the racemic alcohol (**47**) was achieved on a reaction column filled with Novozym 435 in the presence of vinyl acetate as the acyl donor. The targeted (*R*)-bis-THF alcohol was obtained in 46% yield with 99% ee together with the corresponding (*S*)-ester (45% yield, >99% ee). Importantly, the flow process resulted in a distinct intensification of the productivity of all three steps compared to the corresponding batch synthesis.

Enzymes are highly selective systems; however, this extraordinary selectivity frequently involves limited compatibility with regard to various reaction conditions; for example, reagent, solvent and pH tolerance is of key importance in most chemoenzymatic transformations. This often entails significant process design challenges in multistep flow processes comprising biocatalytic key steps. In fact, typical examples on multistep continuous flow syntheses involving biocatalytic transformations rely on a step-by-step process design with individual reaction control, work-up and purification for each segment involved (see, for example, Schemes 19 and 20). Technically more challenging but environmentally and economically more appealing one-flow sequences involving the combination of biocatalysts with divergent downstream chemistries are much less explored.

**Scheme 21:**
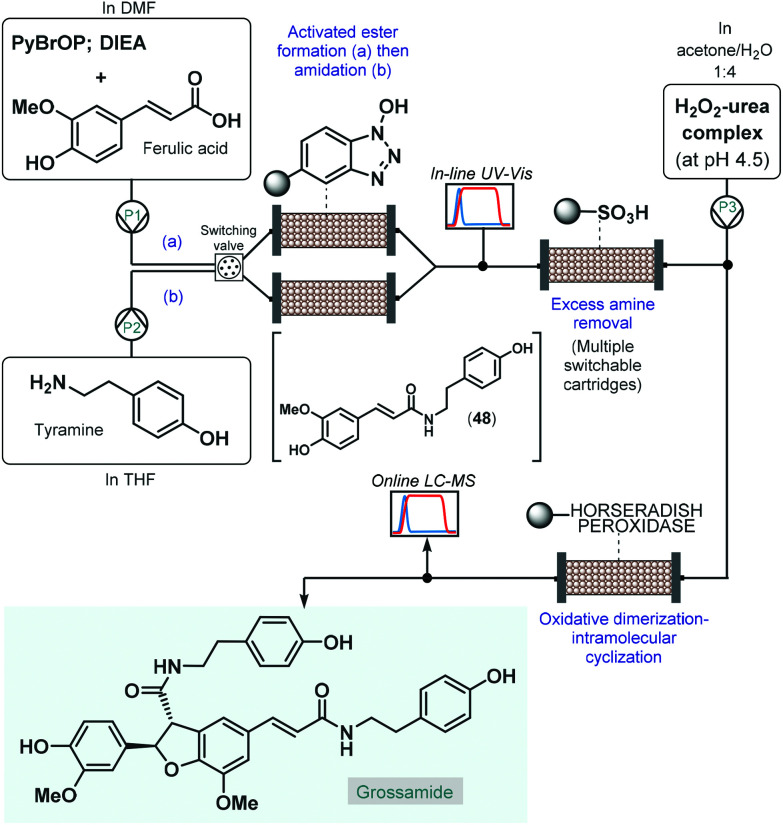
Telescoped continuous flow synthesis of natural product, grossamide.^120^

As a pioneering example, in 2006 Ley and co-workers reported the enantioselective total synthesis of grossamide using an automated flow process.^120^ Although, grossamide is a natural product,^121^ and thus it does not fall into the scope of the present review, the corresponding flow process is surveyed briefly due to its importance. The critical step to introduce asymmetry was an immobilized peroxidase-catalyzed oxidative dimerization–intramolecular cyclization of the key *N*-feruloyltyramine intermediate (**48**), which was achieved *via* peptide coupling of tyramine and ferulic acid (Scheme 21). For synthesis of **48**, the activated ester was formed first in a column packed with PS-supported *N*-hydroxybenzotriazole (HOBt) by pumping a mixture of ferulic acid, bromo-tris-pyrrolidino-phosphonium hexafluorophosphate (PyBrOP) and *N*,*N*-diisopropylethylamine (DIEA) in DMF as solvent. Subsequently, a THF solution of the amine coupling partner, tyramine was directed through the same column to release the activated ester and to yield amide **48**. To achieve continuous operation, two parallel columns were employed; one was continuously loaded with the activated ester as described above, while the other one was used up for the amide formation. From the resulting stream, the excess amine was cleaned up while being passed through a scavenging cartridge containing a sulfonic acid resin. In fact, the system contained multiple switchable scavenging cartridges in order to enable continuous purification as well as simultaneous recovery of the unused tyramine and regeneration of the scavenger. The purified stream was next mixed with H_2_O_2_-urea complex, and grossamide was formed during passage through the final enzyme-loaded column. Importantly, the amide formation step was continuously monitored by means of in-line UV-Vis analysis and the subsequent oxidative dimerization–intramolecular cyclization by means of online LC-MS. By employing real-time data acquisition and feedback-driven automated process control, autonomous operation was achieved while maintaining minimal waste formation under optimum conditions. Despite isolated yield and ee were not disclosed, the reported flow system is claimed to be capable of synthesizing gram quantities of the target compound without manual intervention.

**Scheme 22:**
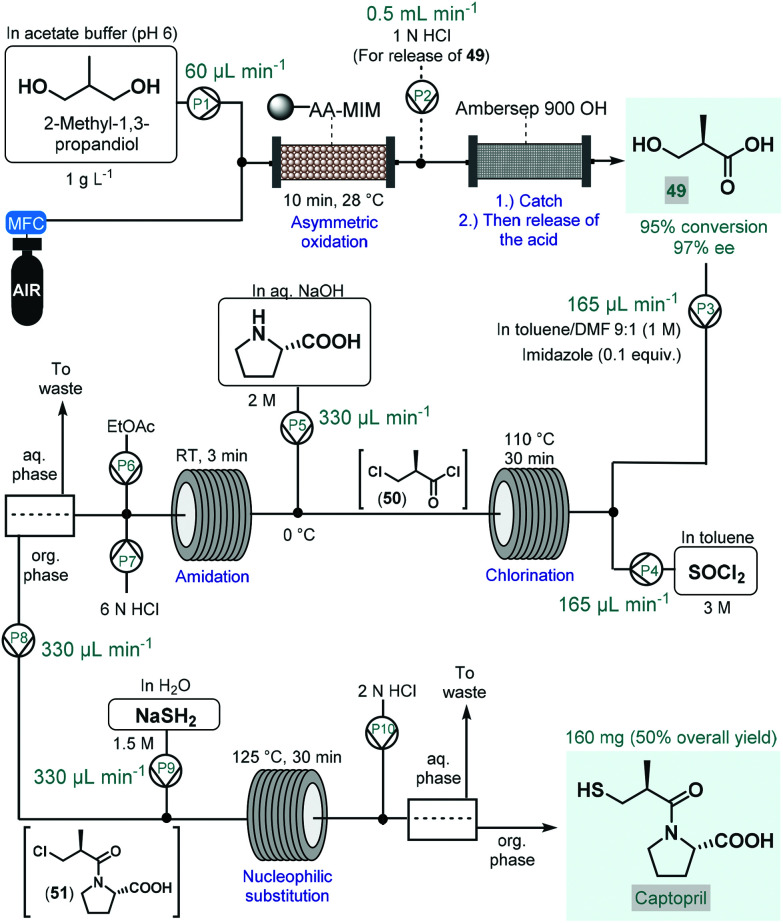
Multistep continuous flow enantioselective synthesis of captopril.^122^ (AA-MIM = whole cells of *Acetobacter aceti* MIM 2000/28).

In 2017, Tamborini and co-workers developed a four-step continuous flow process for the enantioselective synthesis of a widely used antihypertensive drug, captopril (Scheme 22).^122^ The synthesis relied on 2-methyl-1,3-propandiol as a cheap and readily available prochiral starting material, which was transformed into the corresponding (*R*)-methylpropanoic acid-derivative (**49**) *via* regio- and stereoselective chemoenzymatic oxidation, thus eliminating the need for environmentally problematic chemical oxidants. The oxidation was performed by passing a segmented air–liquid flow stream containing the substrate in an acetate buffer through a packed-bed column charged with alginate-immobilized whole cells of *Acetobacter aceti* MIM 2000/28. The system showed a remarkable stability during 10 h of continuous operation and furnished 95% conversion within 10 min residence time with an excellent ee of 97%. The resulting chiral carboxylic acid (**49**) was isolated by trapping on a basic resin (Ambersep 900 OH) followed by release upon washing with aq. HCl solution. For further transformation of **49**, a telescoped three-step sequence was designed without any intermediate purification. First, chlorination in the presence of SOCl_2_ and a catalytic amount of imidazole resulted the corresponding 3-chloro-subsituted acid chloride (**50**) upon passage through a heated reaction coil. For the subsequent amidation, l-proline dissolved in aq. NaOH solution was mixed with the chlorination stream and the resulting mixture was directed through a second coil at room temperature. Following in-line acidification and liquid–liquid separation, the organic phase containing amide **51** was combined with an aq. NaSH solution, and the resulting mixture was submitted to nucleophilic substitution in another coil. Finally, captopril was furnished after in-line acidification, liquid–liquid separation and offline crystallization in 50% overall yield.

**Scheme 23:**
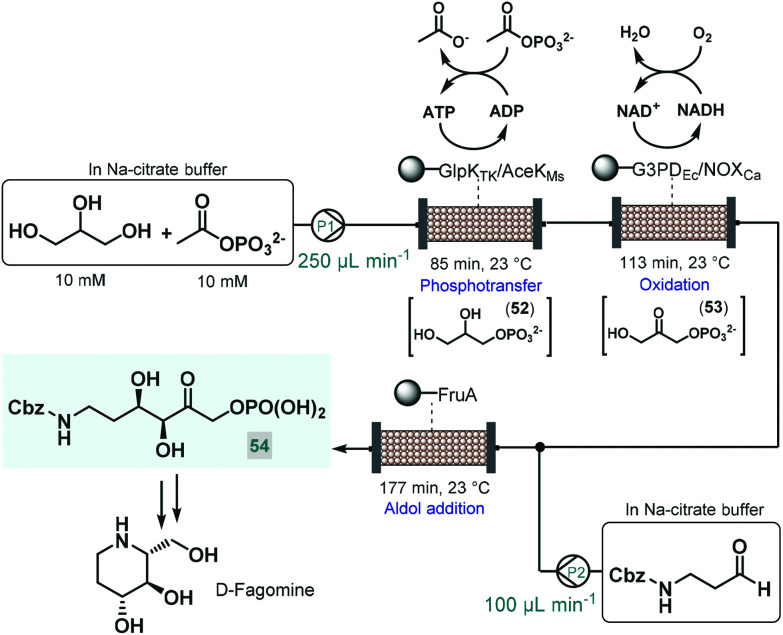
Continuous flow biocatalytic cascade for the asymmetric synthesis of an advanced d-fagomine intermediate.^123^

Recently, an elegant approach was developed by Scott and co-workers for the multistep telescoped chemoenzymatic flow synthesis of an advanced intermediate of the antidiabetic drug, d-fagomine.^123^ Starting from glycerol as substrate, three bioreactors were designed and combined into a series to perform sequential phosphotransfer, oxidation and stereoselective aldol addition (Scheme 23). In order to facilitate waste-minimized operation, each biocatalytic segment comprised an immobilized enzyme, a cofactor recycling unit and a conjugation module. On the basis of batch reaction data, a *Thermococcus kodakarensis* glycerol kinase (GlpK_Tk_) and a *Mycobacterium smegmatis* acetate kinase (AceK_Ms_) were selected for the phosphotransfer, and *Escherichia coli* glycerol-3-phosphate dehydrogenase (G3PD_Ec_) together with the water-forming NADH oxidase from *Clostridium aminovalericum* (NOX_Ca_) for the subsequent oxidation. In these units, the enzyme pairs were cooperatively responsible for the required biocatalytic activity as well as for the recycling and retaining of the bounded cofactor (ATP or NAD^+^). For the third step, a cofactor-independent fructose aldolase (FruA) homologue from *Staphylococcus Carnosus* proved the best. In all three segments, the conjugation module enabled catalyst immobilization *via* strong covalent bonding thus eliminating leaching and preventing contamination of the reaction product. These precisely engineered biocatalysts were packed into glass columns as catalyst beds. During passage through the first column, glycerol was converted to glycerol-3-phosphate (**52**) *via* ATP-dependent regiospecific phosphorylation, which was next transformed into dihydroxyacetone phosphate (**53**) in an NAD^+^-dependent oxidation step. Importantly, ATP and NAD^+^ were retained in the reaction columns due to the presence of the cofactor recycling units. The stream exiting the oxidation column was then mixed with Cbz-protected 3-aminopropanal and the resulting solution was directed through the aldolase column to yield the desired enantiopure (ee > 99%) d-fagomine intermediate (**54**) *via* stereoselective aldol addition. Importantly, the three-step continuous flow biocatalytic sequence proved stable for operation for three consecutive runs (8 h per run) while maintaining product yields between 85% and 90%. A technically similar multienzyme flow approach was reported later by Paradisi and co-workers for the multistep synthesis of l-pipecolic acid, an in important chiral building block for the synthesis of numerous chiral APIs.^124^ The reaction cascade, starting from l-lysine as chiral auxiliary, comprised three sequential steps, a biocatalytic deamination, a spontaneous cyclization and a biocatalytic reduction being orchestrated by two different immobilized enzymes in one reaction column. In this study, the cofactor required for the chemoenzymatic steps was fed in a catalytic amount and was continuously recycled by employing a catch and release strategy. As demonstrated by these examples, one-flow multienzyme approaches hold significant potentials in improving atom as well as step-economy, whilst reducing the configurational complexity of multistep continuous chemoenzymatic flow systems.

**Scheme 24:**
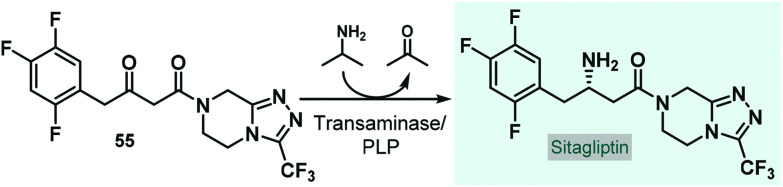
Biocatalytic key step of an end-to-end continuous-flow system designed for the manufacture of sitagliptin.^126,127^

Although, there are recent examples for biocatalytic processes being realized in pilot-plants by employing a series of continuously-stirred tank reactors,^125^ the utilization of continuous flow biocatalytic approaches at a manufacturing-scale is currently very limited. In an interesting recent example, Wang and co-workers designed and modelled an end-to-end continuous-flow system comprising an asymmetric chemoenzymatic key step for the manufacture of the antidiabetic drug, sitagliptin.^126^ On the basis of an earlier study,^127^ an engineered transaminase together with PLP as cofactor was proposed as biocatalyst for the manufacturing-scale asymmetric amination of the corresponding prochiral ketone (**55**) to yield enantiomerically pure sitagliptin (Scheme 24). In order to eliminate leaching issues frequently observed with immobilized enzymes, the authors outlined a process relying on the free enzyme. The designed system consisted of numerous reactor units, mixers, liquid–liquid separators, in-line crystallizers and online analytical devices; and besides the main stream, it comprised several streams to recycle organic solvents as well as the free-enzyme making the whole procedure environmentally sound.

## 5. Summary and outlook

Over the past few years, significant progress has been achieved in the continuous flow asymmetric synthesis of chiral APIs and their advanced intermediates. From the present literature survey, it emerges that organometallic catalysis, organocatalysis as well as biocatalysis are now broadly employed as tools to introduce asymmetry into pharmaceutically relevant substances under continuous flow conditions. It is important to recognize that chiral catalysis acts as a key approach to achieve high selectivity and thus to reduce waste formation, whilst flow chemistry has been proven as an enabling technology potentiating asymmetric reactions towards practical applications. Each catalytic approach exhibits well-defined benefits, but yet disadvantages too. For example, chemoenzymatic approaches are generally ensuring extraordinary enantioselectivity; however, the flexibility of such reactions is limited due to the highly specific chiral recognition. In contrast, typical chemocatalysts offer wider applicability and a more general scope, but in some cases with a lower level of selectivity and/or activity. Another point is that enzymes and chiral organocatalysts enable metal-free conditions, which is especially important in syntheses of pharmaceutically relevant substances.

From environmental and practical points, solid supported chiral catalysts are particularly appealing. Accordingly, in most of the examples studied till date and covered in this review, heterogenized chiral catalysts were preferred in packed-bed systems over soluble catalytic sources. In some cases, leaching and catalyst deactivation was found to reduce reaction efficiency and to hamper long term stability; however, significant efforts have been made to improve catalyst stability either by modifying catalyst-support interactions or by optimization of reaction conditions. In this manner, covalently immobilized catalytic systems bear much lower tendency for leaching, but may require laborious synthetic manipulations to prepare. If catalyst deactivation can be minimized, such packed-bed systems readily facilitate long-runs achieving multigrams of chiral products with simplified isolation. Homogeneous chiral catalytic procedures were also exemplified. These implied no catalyst deactivation issues, but, especially in multistep processes, often involved limited solubility and thus required higher solvent consumption. Moreover, these materials are more difficult to remove, and typically cannot be recycled, thus generating more waste.

Asymmetric reactions involving chiral catalysts were generally conducted in various organic solvents under dilute conditions. Due to solubility issues, environmentally non-acceptable solvents, such as CHCl_3_, CH_2_Cl_2_, dioxane and MTBE had to be used in some instances. Notably, in a few recent examples enantioselective organocatalytic reactions were achieved on synthetically useful scales and productivities under solvent-free conditions. In these instances, the application of continuous flow conditions warranted higher productivities, lower catalyst loadings as well as less waste formation than in the corresponding batch reactions. Aqueous conditions were employed in some of the biocatalytic studies only.

Besides single-step processes, interrupted multistep flow syntheses and combined batch/flow syntheses, telescoped one-flow processes have also emerged for chiral API synthesis. Despite being technically more challenging, these strategies typically involve the generation of less waste due to elimination of intermediate isolation, and tend to be safer than corresponding batch experiments due to *in situ* formation of hazardous reagents and intermediates. Regarding in-line purifications, our literature survey indicates that solid scavengers still tend to dominate, despite the fact that microfluidic extractions or biphasic systems would facilitate truly continuous and scalable operations. Interestingly, PAT-enabled process monitoring has only been scarcely investigated in chiral API syntheses using flow conditions. In the multistep procedures discussed herein, asymmetric key reactions were combined with diverse downstream reactions, such as heterogeneous catalytic hydrogenations, amidations, deprotections. In some cases extreme reaction conditions as well as forbidden chemistries were explored to facilitate clean and direct access to the target compounds. As demonstrated by some recent chemoenzymatic processes, one-flow multi-catalytic approaches may offer considerable benefits in improving atom and step-economy while minimizing waste formation.

The catalytic chiral key reactions to build up asymmetry in the surveyed API syntheses comprised only a limited type of enantioselective transformations, such as various conjugate additions, aldol reactions, aminations, esterifications and oxidations. Therefore, it is foreseen that one of the major directions of future research in this field will be focused towards the broadening of the scope of asymmetric reactions along with novel chiral catalysts for the synthesis of more complex APIs. One of the main driving forces behind the advent of flow chemistry-based techniques for the synthesis of chiral pharmaceuticals is the reduction of the environmental impacts for future manufacturing processes. In this manner, we anticipate that even more emphasis will be placed to the accompanying green metrics and also to the utilization of novel, more effective activation modes (*e.g.*, photo-organocatalysis), that have never been exploited for enantioselective synthesis of APIs under flow conditions. Similarly, a sharper focus is expected on the use of alternative solvents or solvent-free or highly concentrated conditions. Importantly, tech-transfer of such multistep asymmetric flow processes from lab to manufacturing may require significant changes of the current state-of-the-art, therefore novel methodologies and reactor concepts are also anticipated.

Our survey clearly demonstrates that continuous flow enantioselective catalysis has already evolved into a mature field exhibiting a huge potential in advancing future manufacturing processes. However, it is also evident that most examples presented to date possess significant limitations as concerns environmental aspects as well as green metrics. Therefore, as closing remarks, we compiled a list of recommendations as a guidance for future developments in this field under the auspices of green and sustainable chemistry.

• Solvent-free reactions or concentrated solutions should be preferred instead of dilute reaction mixtures.

• If solvent-free conditions cannot be ensured, environmentally-reliable and renewable alternatives should be preferred instead of more conventional harmful solvents.

• Readily reusable heterogeneous catalysts should be preferred with little to no leaching over soluble ones.

• Where possible multistep telescoped processes should be preferred in order to minimize the need for intermediate isolation.

• Truly continuous and scalable in-line purification techniques (*e.g.*, microfluidic extractions) should be preferred over offline approaches.

• Processes should be free of chromatographic purification and should require only minimal amounts of solvents for work-up.

• Processes should be achieved at milder conditions for increased energy efficiency.

• Utilization of PAT-enabled real time analytical data should be stressed to ensure high product quality and to increase process understanding as well as throughput.

• Novel activation modes should be exploited which reduce the number of steps in chiral API synthesis under the auspices of atom- and step-economy.

• Reactions should be highly chemo-, regio- and stereoselective and should rely on the lowest excess of reagents possible in order to minimize byproduct formation and to reduce waste generation.

## Conflicts of interest

There are no conflicts to declare.

## Supplementary Material
